# SPOC1-Mediated Antiviral Host Cell Response Is Antagonized Early in Human Adenovirus Type 5 Infection

**DOI:** 10.1371/journal.ppat.1003775

**Published:** 2013-11-21

**Authors:** Sabrina Schreiner, Sarah Kinkley, Carolin Bürck, Andreas Mund, Peter Wimmer, Tobias Schubert, Peter Groitl, Hans Will, Thomas Dobner

**Affiliations:** Heinrich Pette Institute, Leibniz Institute for Experimental Virology, Hamburg, Germany; University of Michigan, United States of America

## Abstract

Little is known about immediate phases after viral infection and how an incoming viral genome complex counteracts host cell defenses, before the start of viral gene expression. Adenovirus (Ad) serves as an ideal model, since entry and onset of gene expression are rapid and highly efficient, and mechanisms used 24–48 hours post infection to counteract host antiviral and DNA repair factors (e.g. p53, Mre11, Daxx) are well studied. Here, we identify an even earlier host cell target for Ad, the chromatin-associated factor and epigenetic reader, SPOC1, recently found recruited to double strand breaks, and playing a role in DNA damage response. SPOC1 co-localized with viral replication centers in the host cell nucleus, interacted with Ad DNA, and repressed viral gene expression at the transcriptional level. We discovered that this SPOC1-mediated restriction imposed upon Ad growth is relieved by its functional association with the Ad major core protein pVII that enters with the viral genome, followed by E1B-55K/E4orf6-dependent proteasomal degradation of SPOC1. Mimicking removal of SPOC1 in the cell, knock down of this cellular restriction factor using RNAi techniques resulted in significantly increased Ad replication, including enhanced viral gene expression. However, depletion of SPOC1 also reduced the efficiency of E1B-55K transcriptional repression of cellular promoters, with possible implications for viral transformation. Intriguingly, not exclusive to Ad infection, other human pathogenic viruses (HSV-1, HSV-2, HIV-1, and HCV) also depleted SPOC1 in infected cells. Our findings provide a general model for how pathogenic human viruses antagonize intrinsic SPOC1-mediated antiviral responses in their host cells. A better understanding of viral entry and early restrictive functions in host cells should provide new perspectives for developing antiviral agents and therapies. Conversely, for Ad vectors used in gene therapy, counteracting mechanisms eradicating incoming viral DNA would increase Ad vector efficacy and safety for the patient.

## Author Summary

Viruses have acquired functions that target and modulate host cell signaling and diverse regulatory cascades, leading to efficient viral propagation. During the course of productive infection, Ad gene products manipulate destruction pathways to prevent viral clearance or cell death prior to viral genome amplification and release of progeny. Recently, we reported that chromatin formation and cellular SWI/SNF chromatin remodeling processes play a key role in Ad transcriptional regulation. Here, we observe for the first time that SPOC1, identified as a regulator of DNA damage response and chromatin structure, plays an essential role in restricting Ad gene expression and progeny production. This host cell antiviral mechanism is efficiently counteracted by tight association with the major core protein pVII bound to the incoming viral genome. Subsequently, SPOC1 undergoes proteasomal degradation via the Ad E1B-55K/E4orf6-dependent, Cullin-based E3 ubiquitin ligase complex. We also show that other viruses from RNA and DNA families also induce efficient degradation of SPOC1. These analyses of evasion strategies acquired by viruses and other human pathogens should provide important insights into factors manipulating the epigenetic environment to potentially inactivate, or amplify host cell immune responses, since detailed molecular mechanisms and the full repertoire of cellular targets still remain elusive.

## Introduction

DNA viruses require nuclear transport of their genomes to productively infect the host cell and initiate efficient replication. Simultaneously, introduction of viral nucleic acids into the host cell nucleus triggers danger signals, and activates DDR (*DNA damage response*) prior to cell cycle arrest, and apoptosis. Many viruses counteract these regulatory measures in infected cells in order to ensure productive infection, which necessitates proper viral gene expression and adequate progeny synthesis [1].

In line with this, Ad (*Adenoviruses*) have gained functions to modulate DSBR (*double-strand break repair*), apoptosis, cellular gene expression, and host cell immune responses. The incoming viral genome is complexed with core factors and capsid protein VI after endosomal release. Our recent work showed that Ad DNA remains transcriptionally inactive until protein VI mediates activation of the viral E1A promoter by functionally inhibiting chromatin-associated transcription factor Daxx [2]. Ad E1A (*early region 1A protein*) is the first protein expressed after infection, playing an essential role in subsequent transcriptional activation, and induction of cell cycle progression [3].

Recently, E1A was seen to interact with the cellular PML-II isoform, and together this complex elevates transcription from Ad promoters [4]. E1B-55K *(early region 1B 55 kDa protein)* supports efficient viral replication by inhibiting anti-proliferative processes induced by the host cell [5]. However, additional functions of E1B-55K mainly require its interaction with E4orf6 (*early region 4 open reading frame protein 6*). So far, several reports have demonstrated that Ad E4orf6 connects Ad E1B-55K to an E3 ubiquitin ligase complex in the nucleus, containing cellular factors Rbx1/Roc1/Hrt1, Elongin B/C, and either Cullin 2 or Cullin 5 [6]. Recent work has shown that E1B-55K is the substrate recognition unit, while E4orf6 assembles the cellular components, and this functional complex sequesters cellular target proteins into a proteasomal degradation pathway C [6], [7], [8], [9], [10], [11], [12], [13].

Another hurdle to the viability and propagation of DNA viruses is imposed by host DDR and repair machinery. To counter this, several DNA viruses have acquired early viral genes that degrade or redistribute key cellular factors of the repair machinery to protect viral genome integrity [6]. Ad-mediated DDR inhibition by the Ad E1B-55K/E4orf6 E3 ubiquitin ligase complex and E4orf3 (*early region 4 open reading frame protein 3*)-dependent relocalization of the MRN complex into nuclear tracks and cytoplasmic inclusions together block concatemer formation and DNA damage signaling, therefore allowing productive infection and efficient virus growth [14], [15], [16], [17], [18].

The human SPOC1 (*survival-time associated PHD protein in ovarian cancer 1*/PHF13) protein has been identified as a novel regulator of DDR and chromatin structure [19], [20]. The *spoc1* gene is located in chromosomal region 1p36.23, a region with frequent heterozygous deletions implicated in tumor development and progression [21], [22]. Consistent with this, elevated SPOC1 RNA levels in primary and recurrent epithelial ovarian cancers have been associated with decreased survival rates in patients [23]. Moreover, SPOC1 RNA can be detected in most human tissues, with the highest levels in the testis, where it has been exclusively detected in spermatogonia [23], [24].

SPOC1 is a nuclear protein with a PHD (*plant homeodomain*), predicted to bind H3K4me2/3 and to regulate chromatin-specific interactions [20], [25]. In line with this, Kinkley and co-workers observed that SPOC1 is dynamically associated with chromatin and induces chromosome condensation to regulate proper cell division [20]. Particularly, SPOC1 plays a role in radiosensitivity and DNA repair by selective modulation of, and functional cooperation with chromatin modifiers and DDR regulators [19].

There is also evidence that SPOC1 is recruited to DSBs and regulates the kinetics of DSB repair and cellular radiosensitivity. It is proposed that H3K4me2/3-containing chromatin can be converted into more compact chromatin by SPOC1-mediated increase of H3K9 KMTs and H3K9me3. Hence, loss of SPOC1 promotes chromatin decondensation, and is associated with increased levels of DDR transducers and efficient DNA repair. The correlation between SPOC1 protein levels and H3K9me3, as well as expression of several H3K9 KMTs, implicates SPOC1 functions in both chromatin condensation and DDR [19]. In sum, SPOC1 is a multifunctional protein with a additional role in stem cell differentiation, oncogenesis, chromatin structure, and DNA repair processes.

Here, we identified SPOC1 as a novel host restriction factor targeted during viral infection. SPOC1 protein levels decreased in Ad infected cells, which we could attribute to proteasomal degradation mediated by the E1B-55K/E4orf6 E3 ligase complex. Not only did SPOC1 interact with E1B-55K, but also Ad5 E2A-DBP, a marker for nuclear Ad replication sites, as seen by co-immunoprecipitation and immunofluorescence assays. When SPOC1 was overepressed, Ad virus yield, viral DNA synthesis, and viral protein synthesis decreased; reporter gene and chromatin immunoprecipitation assays showed that SPOC1 repressed gene expression at the level of transcription. Intriguingly, interaction with structural viral core protein pVII initially stabilized SPOC1 protein levels before expression of any viral early proteins.

## Results

### Ad5 E3 ubiquitin ligase complex targets SPOC1 for proteasomal degradation

**Figure 1:**
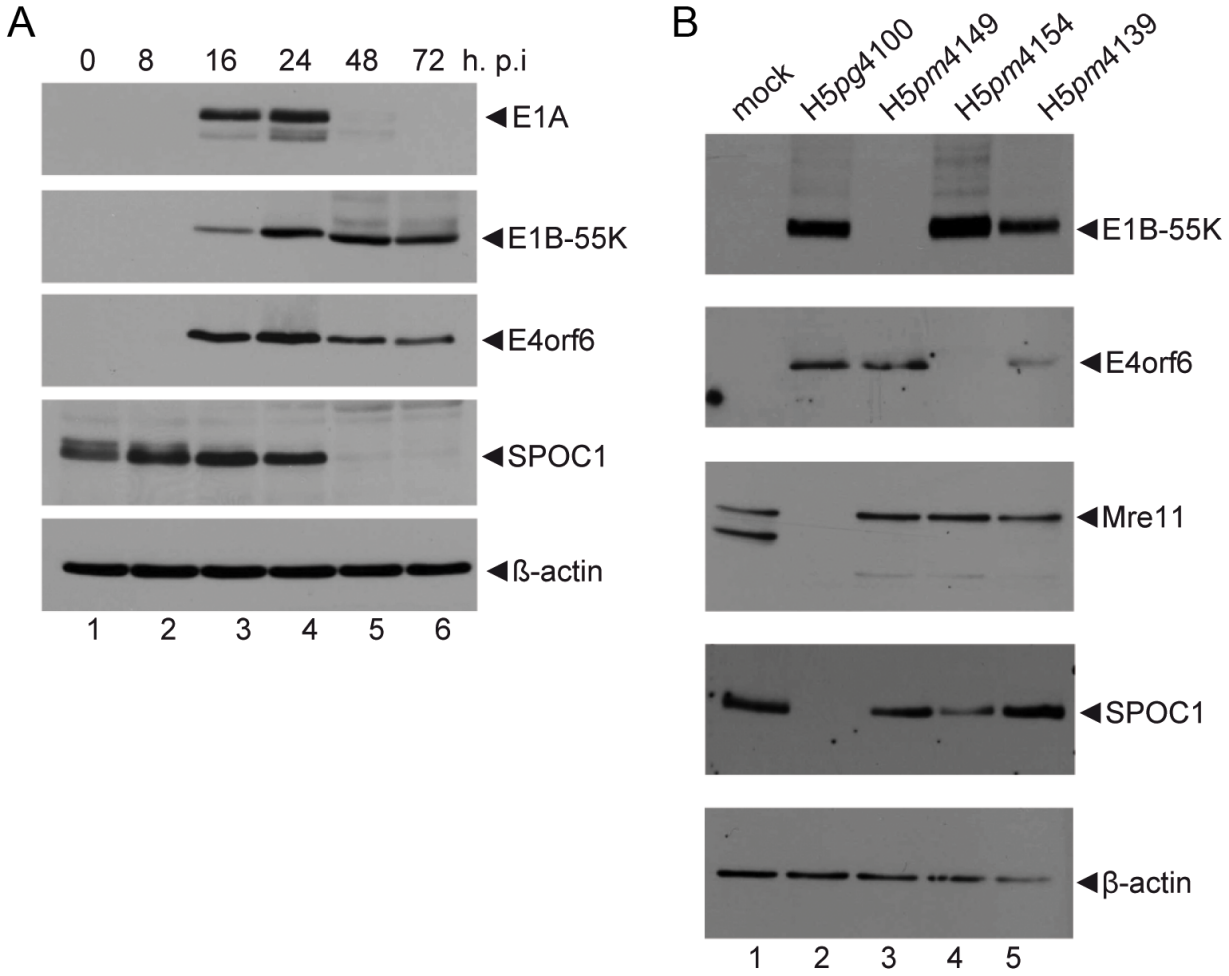
SPOC1 is reduced during Ad infection. H1299 cells were infected with wild type H5*pg*4100 at a multiplicity of 50 FFU per cell. Cells were harvested after indicated time points post infection, total-cell extracts were prepared, separated by SDS-PAGE and subjected to immunoblotting. (A) Immunoblotting using mouse monoclonal antibody M73 (E1A), 2A6 (E1B-55K), RSA3 (E4orf6), rat monclonal SPOC1 antibody and mouse monoclonal antibody AC-15 (β-actin) as a loading control. (B) H1299 cells were infected with wildtype (H5*pg*4100) and mutant viruses (H5*pm*4149, H5*pm*4154, H5*pm*4139) at moi of 50 FFU per cell. Cells were harvested after 48 hours, and immunoblotted using the antibodies above, plus rabbit polyclonal Mre11 antibody.

SPOC1 induces chromosome condensation [20], stimulates cellular gene silencing and influences DDR, which potentially contributes to its transformation potential [19]. Since host DDR prevents viability and propagation of DNA viruses, Ad efficiently targets a multitude of host cell DSB repair regulatory factors in order to promote productive infection [9], [10], [17], [26], [27]. Based on these data, we examined SPOC1 protein levels in infected human cells, and observed SPOC1 levels reproducibly reduced after 24 hours post infection (Fig. 1A). This decrease was even more pronounced at a higher multiplicity of infection (data not shown), and was comparable to the reduction of already known targets of the E1B-55K/E4orf6 E3-ubiquitin ligase complex [6].

To discover whether SPOC1 is a new host cell substrate of the Ad5 E3 ubiquitin ligase complex, we determined SPOC1 protein concentrations in wild type (H5*pg*4100), and mutant-virus infected cells lacking either E1B-55K (H5*pm*4149) or E4orf6 (H5*pm*4154) (Fig. 1B). As anticipated, SPOC1 was dramatically reduced in cells infected with the wild type virus H5*pg*4100 (Fig. 1B, lane 2), whereas the cellular protein accumulated to levels comparable to non-treated cells in infected cells lacking E1B-55K (H5*pm*4149; Fig. 1B, lane 3). Similarly, SPOC1 was not reduced in cells infected with the E4orf6-minus virus mutant H5*pm*4154 (Fig. 1B, lane 4). We further examined SPOC1 protein levels in H1299 cells infected with an E4orf6 virus mutant (H5*pm*4139) carrying point mutations in the BC Box that abrogate the formation of the Ad5 E3 ubiquitin ligase complex, by inhibition of E4orf6 binding to Elongins B and C. Again activity of the E1B-55K/E4orf6 E3 ligase complex apparently played a role in reducing SPOC1 protein concentrations (Fig. 1B, lane 5). Consistent with previous publications, Mre11 was not degraded during lytic infection with the BC Box virus mutant H5*pm*4139 (Fig. 1B, lane 5). Our results confirm that the formation and ligase activity of the E1B-55K/E4orf6 ubiquitin complex are essential to reduce SPOC1 protein levels.

**Figure 2:**
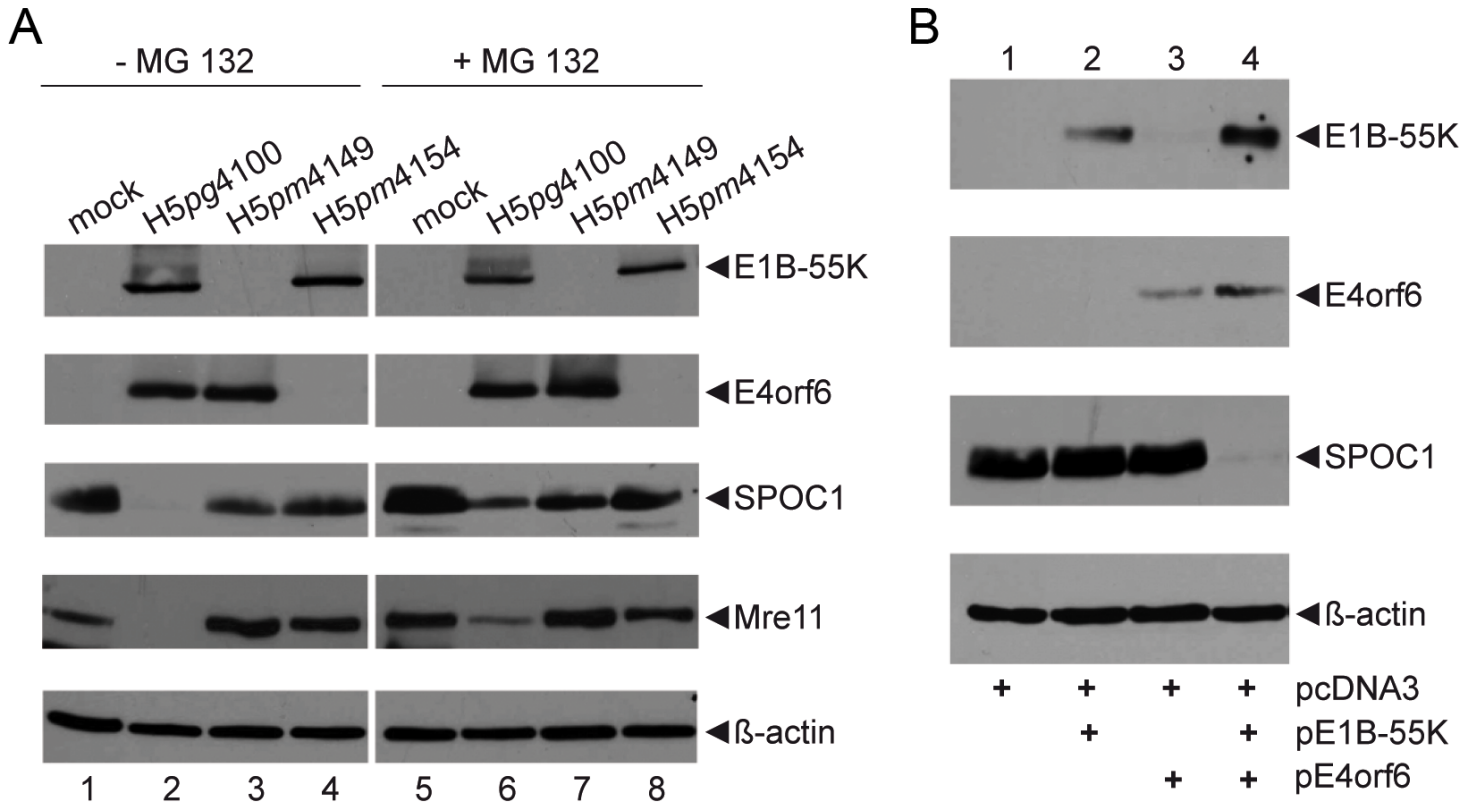
Proteasomal degradation of SPOC1 by the E1B-55K/E4orf6 E3 ubiquitin ligase complex. (A) H1299 cells were infected with wildtype (H5*pg*4100) and mutant viruses (H5*pm*4149, H5*pm*4154) at moi of 50 FFU per cell. Cells were treated for six hours with proteasome inhibitor (+MG 132), before total-cell extracts were prepared 48 h p.i.. Proteins were separated by SDS-PAGE and subjected to immunoblotting using mouse monoclonal antibody 2A6 (E1B-55K), RSA3 (E4orf6), rat monclonal SPOC1 antibody and mouse monoclonal antibody AC-15 (β-actin) as a loading control. (B) H1299 cells were transfected with pcDNA3-derived plasmids expressing wildtype E1B-55K, E4orf6, or a combination of both. Cells were harvested 48 hours post infection. Total cell extracts were prepared and specific proteins were immunoblotted as described in A.

To confirm that we had identified SPOC1 as a novel target of virus-induced proteasomal degradation via E1B-55K/E4orf6-dependent E3 ligases, we treated infected cells with the proteasome inhibitor MG-132 (Fig. 2A). Functionally inhibiting host cell proteasomes abolished Mre11 and SPOC1 reduction seen in wild type infected cells, supporting the role of ubiquitin proteasome system in SPOC1 degradation.

We finally validated our findings by analyzing the levels of SPOC1 protein in transfected cells (Fig. 2B). Expression of E1B-55K alone (Fig. 2B, lane 2) or E4orf6 alone (Fig. 2B, lane 3) had no effect on steady-state concentrations of endogenous SPOC1 protein, while expression of both E1B-55K and E4orf6 (Fig. 2B, lane 4) diminished SPOC1 steady-state concentrations to levels similar to virus-infected cells (Fig. 1 and 2A).

### Ad5 E1B-55K binds and induces cytoplasmic relocalization of SPOC1

**Figure 3:**
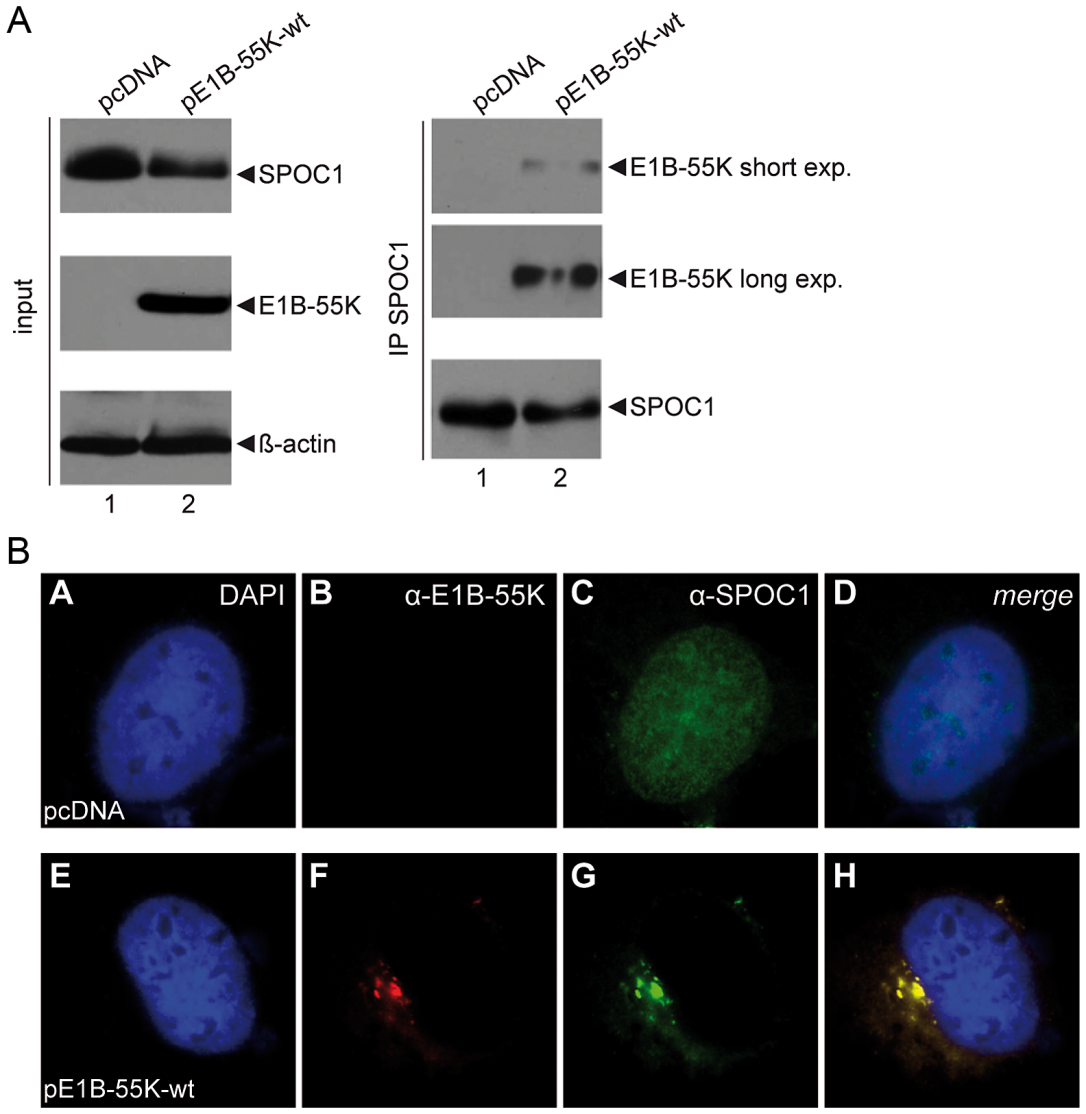
SPOC1 is a novel interaction partner of viral E1B-55K protein. (A) H1299 cells were transfected with empty control plasmid or wild type E1B-55K (pE1B-55K-wt). 24 hours post transfection total cell extracts were prepared. E1B-55K was immunoprecipitated using rabbit polyclonal SPOC1 ab. Proteins were separated on 10% SDS-PAGE and visualized by immunoblotting. Input levels of total cell lysates and co-precipitated proteins were detected using monoclonal antibody 2A6 (E1B-55K), SPOC1-specific rat monoclonal antibody and mouse monoclonal antibody AC-15 (β-actin) as a loading control. (B) H1299 cells were transfected with plasmid constructs encoding wild type E1B-55K. After methanol fixation, the cells were labeled with mouse monoclonal antibody 2A6 (E1B-55K) and SPOC1 specific rat monoclonal antibody. Primary antibodies were detected with Texas Red and FITC conjugated secondary antibody. Representative staining patterns are shown. Nuclei are visualized by DAPI staining (magnification ×7600).

Since E1B-55K is the substrate recognition unit of the SCF-like E3 ubiquitin ligase, we next tested whether E1B-55K interacts with the endogenous SPOC1 protein. As anticipated, in E1B-55K-transfected human H1299 cells E1B-55K co-immunoprecipitated with SPOC1-specific antibody, revealing an interaction between these factors (Fig. 3A, lane 2). No E1B-55K signal was observed in the corresponding negative controls (Fig. 3A, lane 1).

The next question was whether E1B-55K interferes with the intracellular localization of SPOC1. Consistent with previous findings, immunofluorescence analysis in E1B-transfected human cells revealed that wild type E1B-55K protein localizes in the cytoplasm, mostly concentrated in perinuclear bodies Fig. 3B, panel F, H; [28], [29], [30], [31], [32], [33], [34]. In contrast to the diffuse nuclear localization in non-treated cells (Fig. 3B, panel C, D), SPOC1 was completely sequestered into the E1B-containing aggregates in the presence of the viral protein (Fig. 3B, panel G, H).

### SPOC1 is recruited into Ad replication centers

**Figure 4:**
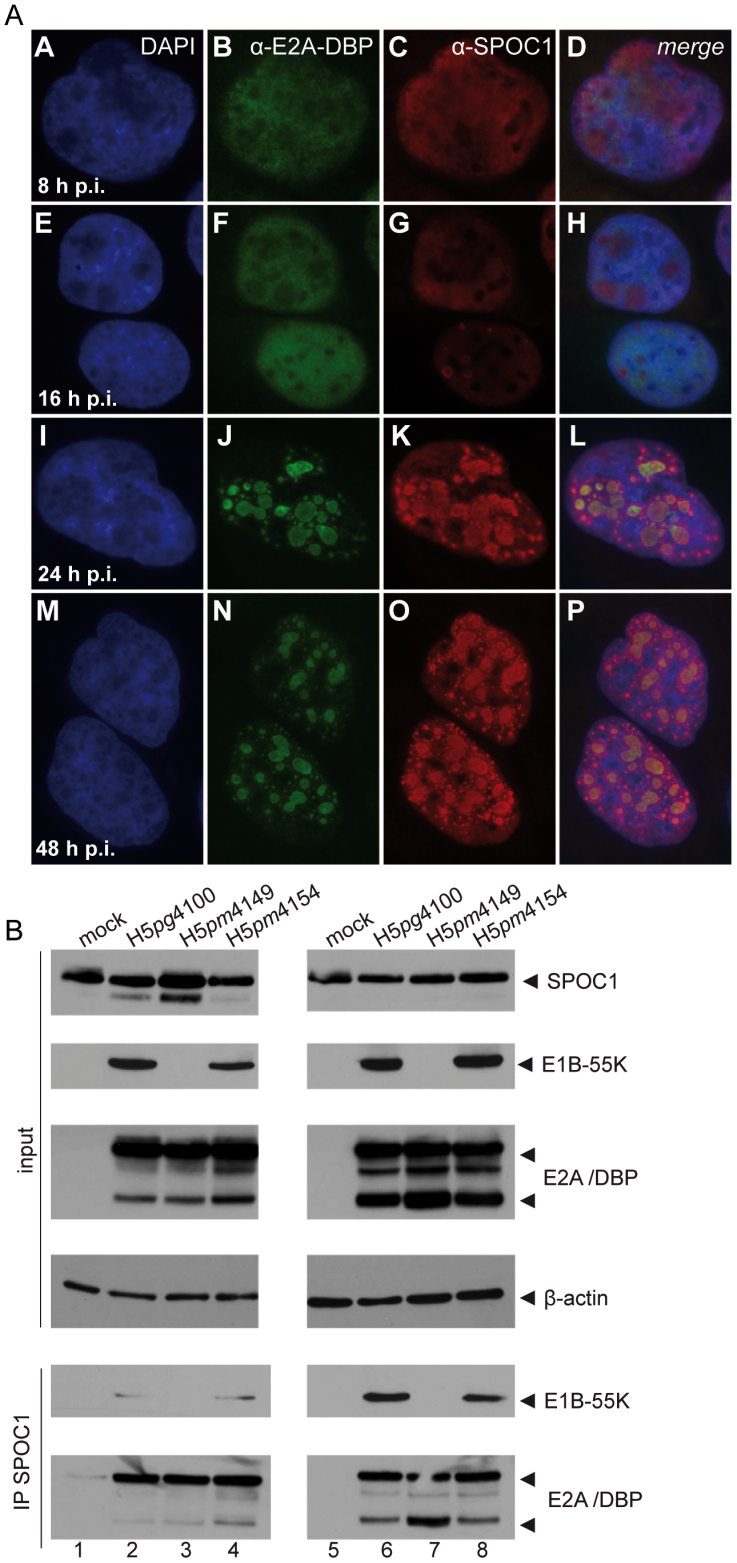
SPOC1 is recruited to Ad replication centers during infection. (A) Human DLD1 cells, which stably overexpress SPOC1 after doxycyclin treatment to overcome degradation of the cellular factor, were infected with wildtype H5*pg*4100 at a moi of 10 FFU per cell; throughout the experiment the cells were grown in media supplied with doxycyclin. After indicated time points, cells were fixed with methanol and labeled with E2A/DBP mouse mab B6-8 and SPOC1-specific rat monoclonal antibody. Primary antibodies were detected with Texas Red or FITC conjugated secondary antibody. Representative staining patterns are shown. Nuclei are visualized by DAPI staining (magnification ×7600). Additionally, merge panels show colocalization of the indicated proteins. (B) DLD1 (left panel) and U2OS (right panel) cells, which stably overexpress SPOC1 after doxycyclin treatment were infected with wildtype (H5*pg*4100) and mutant viruses (H5*pm*4149, H5*pm*4154) at moi of 50 FFU per cell. Cells were treated with doxycyclin 12 hours before infection and throughout the experiment to stably induce SPOC1 expression, before total cell extracts were prepared 24 hours post infection. E1B-55K and E2A/DBP were immunoprecipitated using rabbit polyclonal SPOC1 antibody. Proteins were separated on 10% SDS-PAGE and visualized by immunoblotting. Input levels of total-cell lysates and co-precipitated proteins were detected using monoclonal antibody 2A6 (E1B-55K), B6-8 (E2A/DBP), SPOC-1-specific rat monoclonal antibody, and mouse monoclonal antibody AC-15 (β-actin) as a loading control.

To gain better understanding of SPOC1 role during Ad infection, we investigated the subcellular localization of SPOC1 in infected human DLD1 cells, which stably overexpress SPOC1 after doxycyclin treatment to overcome degradation of the cellular factor (Fig. S1). In mock-infected cells, SPOC1 is diffusely distributed inside the nucleus in approximately 90% of the cells investigated (n = 50; data not shown). When monitored together with Ad5 E2A-DBP, a marker for Ad replication sites in the nucleus, at 24 to 48 hours post infection SPOC1 colocalized with sites of viral replication in approximately 60% of cells (n = 50; Fig. 4A, panels D, H, L and P).

We confirmed our observation by monitoring SPOC1/E2A-DBP association using immunoprecipitation analysis after wild type and mutant virus infection in DLD1 and U2OS cells stably overexpressing SPOC1 after doxycyclin treatment (Fig. 4B). As expected, E2A-DBP co-immunoprecipitated with SPOC1-specific antibody, revealing an interaction in all infected SPOC1-overexpressing human cells (Fig. 4B, lanes 2, 3, 4, 6, 7, 8), whereas no signal was obtained in the corresponding negative controls (Fig. 4B, lanes 1 and 5). We also were able to confirm the SPOC1/E1B-55K interaction within the infected SPOC1-induced cells. To further test specificity of the SPOC1 antibody used in all of the coIPs, we tested SPOC1 binding after proteasomal degradation of SPOC1 (Fig. S2). Therefore, we infected H1299 cells with Ad5 wild type virus (H5*pg*4100) and virus mutants depleted for either E1B-55K (H5*pm*4149) or E4orf6 (H5*pm*4154) expression. After 24 and 48 hors post infection, total cell extracts were prepared. E1B-55K and E2A/DBP were immunoprecipitated using rabbit polyclonal SPOC1 antibody. Proteins were separated on 10% SDS-PAGE and visualized by immunoblotting. Input levels of total-cell lysates and co-precipitated proteins were detected using monoclonal antibody 2A6 (E1B-55K), B6-8 (E2A/DBP), SPOC-1-specific rat monoclonal antibody, and mouse monoclonal antibody AC-15 (β-actin) as a loading control. As expected, E2A-DBP co-immunoprecipitated with SPOC1-specific antibody (Fig. S2, lanes 3, 5–8), only revealing binding in infected cells, where SPOC1 could not be degraded due to the time point (Fig. S2, lane 3) or the absence of either E1B-55K (Fig. S2, lanes 5 and 6) or E4orf6 (Fig. S2, lanes 7 and 8). We also were able to confirm the specific SPOC1/E1B-55K interaction within the infected cells expressing SPOC1 and E1B-55K (Fig. S2, lanes 3, 7 and 8). Together these data improved our co-immunoprecpitation analysis and reassured specificity of the SPOC1 antibody usd in these experiments.

Together, our results show that the cellular factor SPOC1 associates with E2A-DBP within Ad5 replication compartments in virus-infected human cells. This novel observation prompted us to investigate whether SPOC1 is linked to Ad transcriptional regulation during productive infection. Such recruitment to nuclear sites associated with viral genome replication suggests that SPOC1 is involved in regulating Ad5 gene expression.

### SPOC1 overexpression reduces Ad gene expression

**Figure 5:**
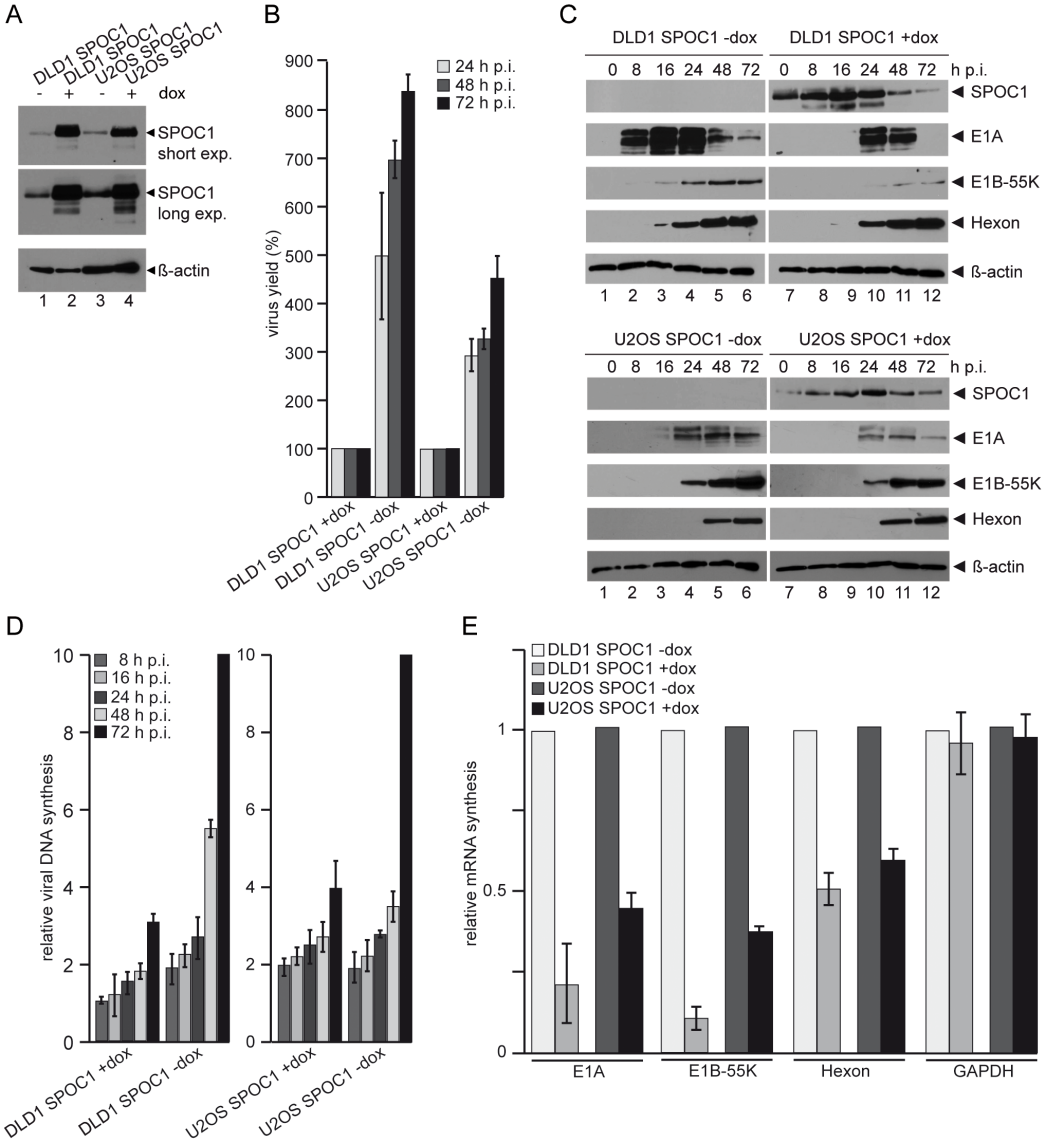
SPOC1 overexpression represses Ad5 gene expression. (A) To validate the model system, DLD1 or U2OS cells were treated with doxycyclin 12 hours before infection to induce SPOC1 expression; throughout the experiment the cells were grown in media supplied with doxycyclin. Cell extracts were subjected to 10% PAGE and immunoblotting using rat monclonal SPOC1 antibody and mouse monoclonal antibody AC-15 (β-actin) as a loading control. (B) DLD1/U2OS cells were infected with wild type H5*pg*4100 at a multiplicity of 50 FFU per cell. Viral particles were harvested at the indicated time-points after infection and virus yield was determined by quantitative E2A-72K immunofluorescence staining on HEK293 cells. The results represent the average from three independent experiments and were normalized to values for particle synthesis in SPOC1 induced cells infected with wild type H5*pg*4100. (C) DLD1/U2OS cells were infected with wild type H5*pg*4100 at a multiplicity of 50 FFU per cell. Total-cell extracts were prepared at the indicated times post infection. Proteins were separated by 10% SDS-PAGE, and immunoblotted with mouse monoclonal antibody M73 (E1A), 2A6 (E1B-55K), anti-Ad5 rabbit polyclonal serum L133, rat monclonal SPOC1 antibody and mouse monoclonal antibody AC-15 (β-actin) as a loading control. (D) DLD1/U2OS cells were infected with wild type H5*pg*4100 at a multiplicity of 50 FFU per cell. Total cell extracts were prepared and treated with proteinase K. PCR was performed using E1B-specific primers (E1B-fw 3′-CGC GGG ATC CAT GGA GCG AAG AAA CCC ATC TGA GC-5′; E1B-rev 3′-CGG TGT CTG GTC ATT AAG CTA AAA-5′). The same amounts of PCR product were separated on an analytic agarose gels (1%) and quantification was achieved with the *Gene Snap Software* (*Syngene*). The results shown represent the averages from three independent experiments. (E) DLD1/U2OS cells were infected with wild type H5*pg*4100 at a multiplicity of 50 FFU per cell. 24 h p.i. total RNA was extracted, reverse transcribed and quantified by RT-PCR using primers specific for E1A (E1A fwd: 5′ GTGCCCCATTAAACCAGTTG 3′; E1A rev: 5′ GGCGTTTACAGCTCAAGTCC 3′), E1B-55K (E1B fwd: 5′-GAGGGTAACTCCAGGG TGCG-3′; E1B rev: 5′-TTTCACTAGCATGAAGCAACCACA-3′), hexon (hexon rev: 5′-GAACGGTGTGCGCAGGTA-3′; hexon fwd 5′-CGCTGGACATGACTTTTG AG-3′) and GAPDH (GAPDH fwd:5′-ACCACAGTCCATGCCATCAC-3′ rev:5′-TCCACCACCCTGTTGCTGTA-3′). Data were normalized to 18S rRNA levels (18S rRNA fwd: 5′-CGGCTACCACATCCAAGGAA-3′; 18S rRNA rev: 5′-GCTGGAATTACCGCGGCT-3′). Values correspond to the mean of triplicates and error bars indicate the standard error of the mean.

To better analyze the role of SPOC1 during Ad5 infection, we performed experiments in SPOC1-inducible human colon carcinoma (DLD1) and osteosarcoma (U2OS) cell lines, which express exogenous SPOC1 after doxycyclin (dox) treatment. Prior to our analysis, we investigated SPOC1 protein expression in the absence and presence of dox (Fig. 5A). Additionally, proliferation of the SPOC1 overexpressing cells was quantified, revealing no significant difference in comparison to the uninduced controls (Fig. S1).

To assess the effect of SPOC1 on overall virus growth, we determined total virus yield after SPOC1 induction in DLD1 cells (Fig. 5B). SPOC1 overexpression reduced progeny production seven- (48 h p.i.) to eight-fold (72 h p.i.) compared to non-treated DLD1 control cells (Fig. 5B). When we analyzed Ad5 progeny production in SPOC1 inducible U2OS cells we observed similar effects (Fig. 5B).

These results suggest that SPOC1 mediates repressive effects during Ad5 infectious cycle. To further validate this hypothesis, expression of viral early and late proteins was monitored at different time points after infection (Fig. 5C). Consistent with Ad5 progeny production, protein synthesis was inefficient in SPOC1 overexpressing DLD1 and U2OS cells (Fig. 5C). Particularly expression of E1A was affected, since it was substantially higher in non-treated DLD1 (Fig. 5C, upper panel lanes 1–6) and U2OS (Fig. 5C, lower panel lanes 1–6). Similar effects were observed when monitoring viral DNA synthesis in infected cells. As with Ad5 progeny production (Fig. 5D), DNA synthesis was less efficient in SPOC1 induced cells than in uninduced cells (Fig. 5D). Next we investigated whether Ad transcription is negatively regulated by SPOC1 expression. Therefore, we analyzed whether early and late viral mRNA levels are affected by SPOC1 overexpression in Ad wild type virus infected cells. We observed that viral early E1A and E1B mRNA production is lower in SPOC1-induced cells, compared to cells lacking treatment with doxycyclin (Fig. 5E). Similar results were obtained for hexon mRNA expression, suggesting either an impact of enhanced synthesis of early gene products or direct repression of the late promoter (Fig. 5E). Moreover, we tested cellular mRNA levels to exclude non-specific repression of gene expression by SPOC1 expression affecting cellular RNA Pol II transcription (Fig. 5E). Our results show similar GAPDH mRNA synthesis in the cells tested, indicating that SPOC1 expression does not reflect an overall reduction in cellular RNA pol II transcription.

**Figure 6:**
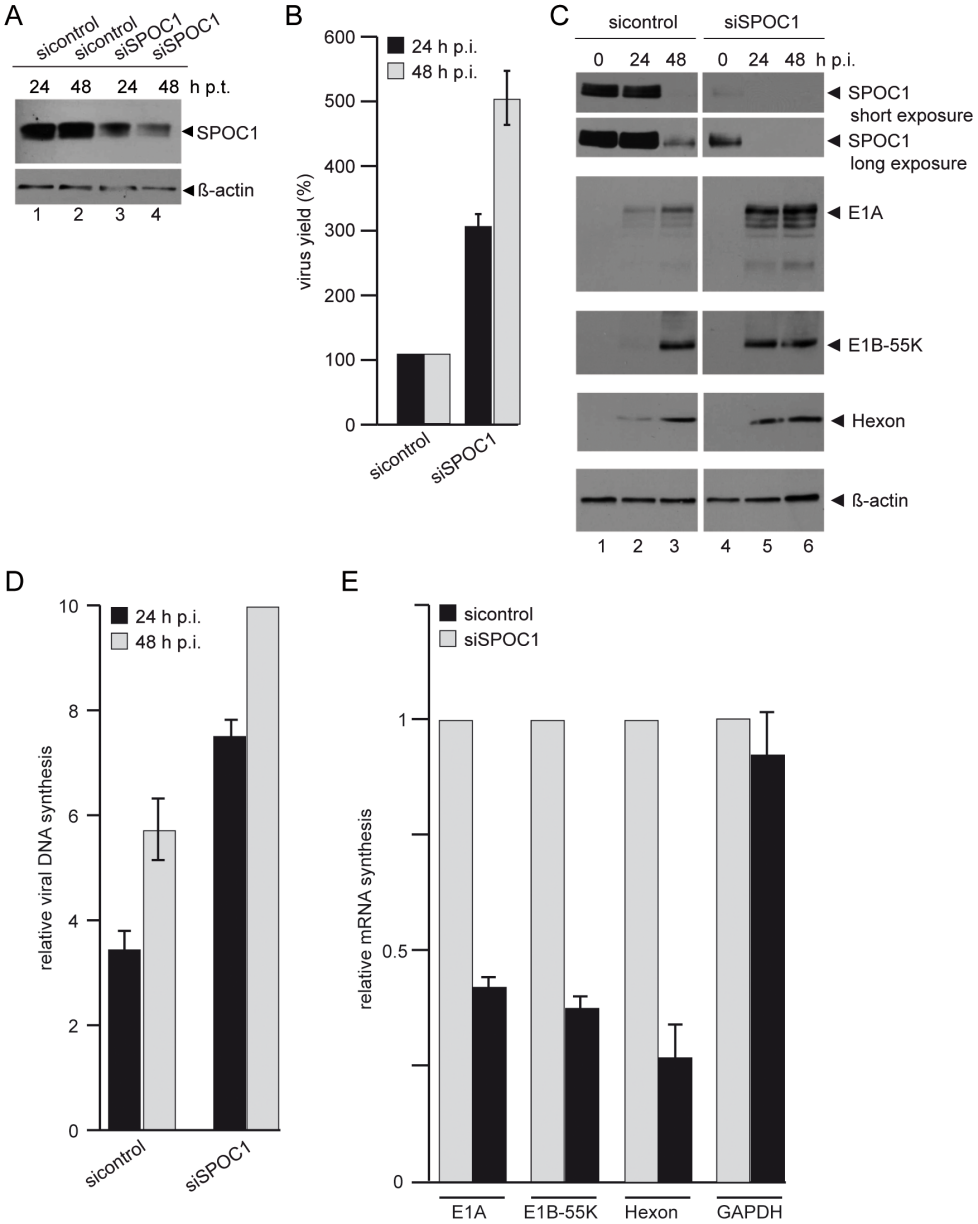
SPOC1 depletion enhances Ad5 gene expression. (A) H1299 cells were transfected with siRNA constructs against SPOC1 (si768, 5′-UCA CCU GUC CUG UGC GAA AdTdT-3′) and control siRNA (5′-AGG UAG UGU AAU CGC CUU GdTdT-3′). Cells were harvested at 24 and 48 hours post transfection. Total cell extracts were prepared and proteins were separated by SDS-PAGE and subjected to immunoblotting using SPOC1-specific rat monoclonal ab. β-actin was included as a loading control. (B) H1299 cells were treated with indicated siRNAs to induce SPOC1 depletion and infected 24 hours post transfection with wild type H5*pg*4100 at a multiplicity of 50 FFU per cell. Viral particles were harvested at the indicated time-points after infection and virus yield was determined by quantitative E2A-72K immunofluorescence staining on HEK293 cells. The results represent the average from three independent experiments. (C) H1299 cells were infected with wild type H5*pg*4100 at a multiplicity of 50 FFU per cell. Total-cell extracts were prepared at the indicated times post infection. Proteins were separated by 10% SDS-PAGE, and immunoblotted with mouse monoclonal antibody M73 (E1A), 2A6 (E1B-55K), anti-Ad5 rabbit polyclonal serum L133, rat monclonal SPOC1 antibody and mouse monoclonal antibody AC-15 (β-actin) as a loading control. (D) H1299cells were infected with wild type H5*pg*4100 at a multiplicity of 50 FFU per cell. Total cell extracts were prepared and treated with proteinase K. PCR was performed using E1B-specific primers (E1B-fw 3′-CGC GGG ATC CAT GGA GCG AAG AAA CCC ATC TGA GC-5′; E1B-rev 3′-CGG TGT CTG GTC ATT AAG CTA AAA-5′). The same amounts of PCR product were separated on an analytic agarose gels (1%) and quantification was achieved with the *Gene Snap Software* (*Syngene*). The results shown represent the averages from three independent experiments. (E) H1299 cells were infected with wild type H5*pg*4100 at a multiplicity of 50 FFU per cell. 24 h p.i. total RNA was extracted, reverse transcribed and quantified by RT-PCR using primers specific for E1A (E1A fwd: 5′ GTGCCCCATTAAACCAGTTG 3′; E1A rev: 5′ GGCGTTTACAGCTCAAGTCC 3′), E1B-55K (E1B fwd: 5′-GAGGGTAACTCCAGGG TGCG-3′; E1B rev: 5′-TTTCACTAGCATGAAGCAACCACA-3′), hexon (hexon rev: 5′-GAACGGTGTGCGCAGGTA-3′; hexon fwd 5′-CGCTGGACATGACTTTTGAG-3′) and GAPDH (GAPDH fwd:5′-ACCACAGTCCATGCCATCAC-3′ rev:5′-TCCACCACCCTGTTGCTGTA-3′). Data were normalized to 18S rRNA levels (18S rRNA fwd: 5′-CGGCTACCACATCCAAGGAA-3′; 18S rRNA rev: 5′-GCTGGAATTACCGCGGCT-3′). Values correspond to the mean of triplicates and error bars indicate the standard error of the mean.

To validate these observations and to exclude non-specific impact of the overexpression studies, we depleted SPOC1 expression by transient knock-down with siRNA (Fig. 6A) and analyzed Ad5 replication. Virus growth was enhanced three-fold (24 h p.i.) to five-fold (48 h p.i.) in the absence of SPOC1, compared to control cells (Fig. 6B). Consistent with data observed in DLD1 and U2OS cells, early and late viral protein synthesis was more efficient in SPOC1-depleted cells (Fig. 6C). DNA synthesis was monitored and showed two-fold more efficient synthesis of viral DNA in SPOC1-depleted cells compared to the control cells (Fig. 6D). To further investigate a role of SPOC1 in Ad transcription and viral mRNA synthesis, we analyzed whether early and late viral mRNA levels are affected by the absence of SPOC1 expression in infected cells (Fig. 6E). We observed that viral early E1A and E1B mRNA production is two-fold reduced in SPOC1 expressing cells, compared to cells lacking this cellular transcription factor. Results obtained for hexon mRNA expression showed three-fold difference in viral mRNA expression (Fig. 6E). As shown above, we did not observe altered GAPDH mRNA levels, indicating that SPOC1 depletion does not affect cellular transcription in general (Fig. 6E).

To exclude that SPOC1 expression impacts on virus entry, we analyzed whether capsid protein VI from incoming Ad capsids show altered accumulation in the host cell nuclear fraction. Using nucleo-cytoplasmic fractionation, we observed rapid protein VI accumulation in the nuclear fraction after infection of U2OS control cells and cells treated with doxycyclin to induce SPOC1 expression (Fig. S3).

Together, these results indicate that cellular factor SPOC1 is a potent repressor of Ad5 growth and thus represents a novel host cell restriction factor during Ad productive infection.

### SPOC1 represses Ad promoter activity

**Figure 7:**
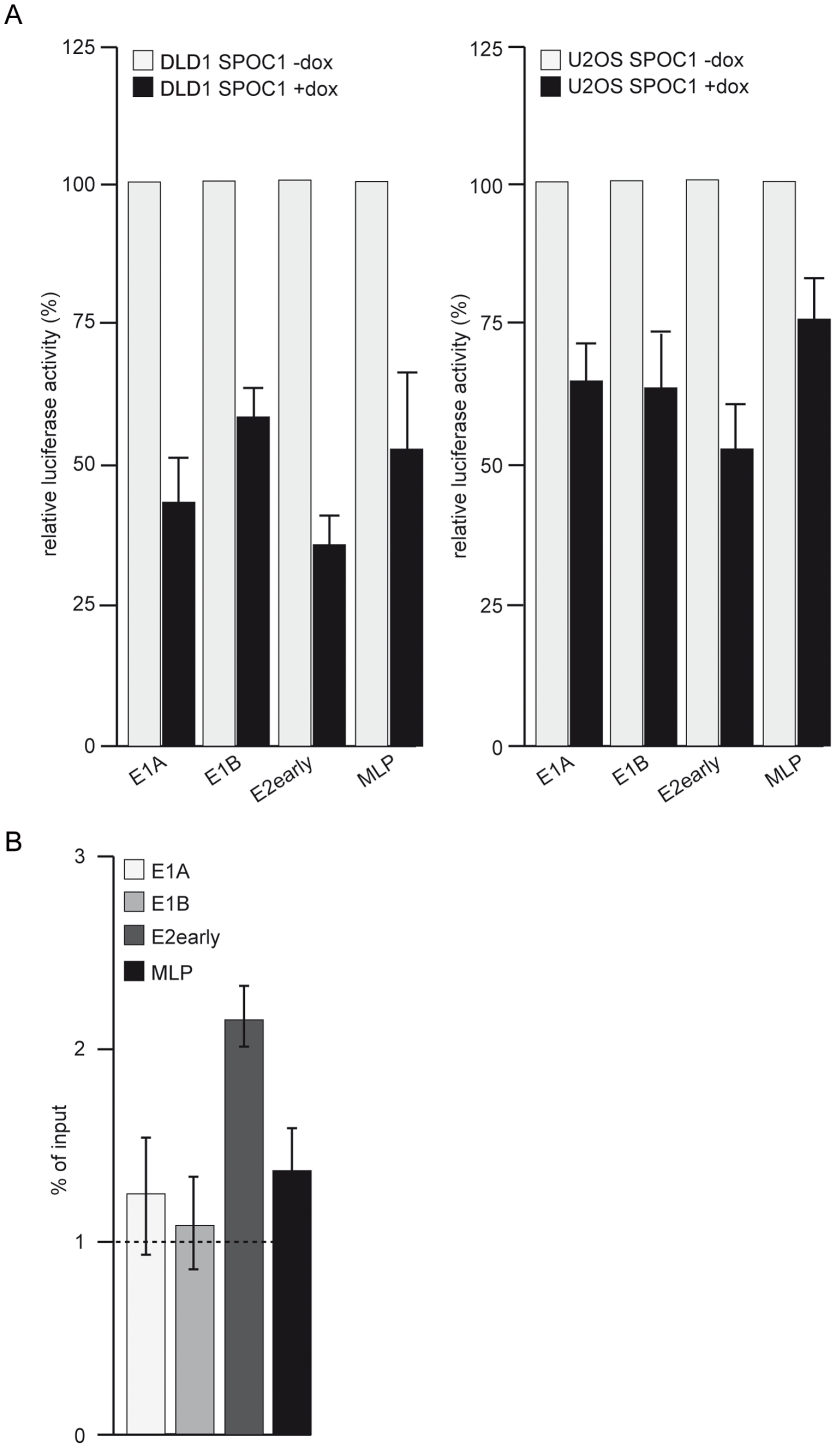
SPOC1 represses viral transcription. DLD1 or U2OS cells were treated with doxycyclin for 12(A) Transfection with luciferase reporter plasmids under Ad promoter control (E1A, E1B, E2early, MLP). 48 h after transfection, samples were lysed and absolute luciferase activity was measured. All samples were normalized for transfection efficiency by measuring *Renilla*-luciferase activity. Activity of empty promotor constructs, E1A, E1B, E2early and MLP promoters in non-treated and non-overxpressing cells was normalized to 100%. Mean and STD are from three independent experiments. (B) After infection with H5*pg*4100 Ad5 wildtype at moi of 20 FFU/cell. 24 hours post infection SPOC1-induced cells were fixed with formaldehyde and analyzed by ChIP assays (see Material and Methods). The average C_t_-value was determined from triplicate reactions and normalized against non-specific IgG controls with standard curves for each primer pair. The y-axis indicates the percentage of immunoprecipitated signal from the input ( = 100%). The dotted line highlights values above 1% of input, commonly stated as significant chromatin/protein binding.

Due to the fact that SPOC1 is a chromatin bound protein, we next questioned whether SPOC1-dependent Ad restriction occurs on the transcriptional level. To assess this, we performed reporter gene assays with luciferase expression vectors under the control of specific Ad promoters in SPOC1-induced and non-induced DLD1 and U2OS cells. In the absence of other viral factors, SPOC1 was able to repress luciferase expression from all the viral promoters tested (Fig. 7A).

To exclude non-specific repression of gene expression, we investigated whether we can detect SPOC1-dependent effects on the cellular E2F-dependent H2A promoter (Fig. S4). In contrast to the viral reporter constructs, co-expression of SPOC1 did not affect transcription from the cellular promoter (Fig. S4, lanes 3 and 4). The inhibitory effect of SPOC1 expression is specific at least for the promoter sequences tested so far.

To further demonstrate direct association of SPOC1 with Ad promoter sequences, we performed chromatin immunoprecipitation assays from wild type virus infected cells, using SPOC1-specific monoclonal antibody, unrelated IgG control antibody and Ad promoter-specific oligonucleotides (Fig. 7B). The results show that SPOC1 is associated with E1A, E1B, E2early and MLP Ad promoters in infected cells (Fig. 7B). Conclusively, these results indicate that SPOC1 is a component of host cell antiviral mechanisms, playing an important role in the Ad gene expression program through transcriptional repression of viral promoters.

### Functional cooperation between SPOC1 and Ad core protein VII

**Figure 8:**
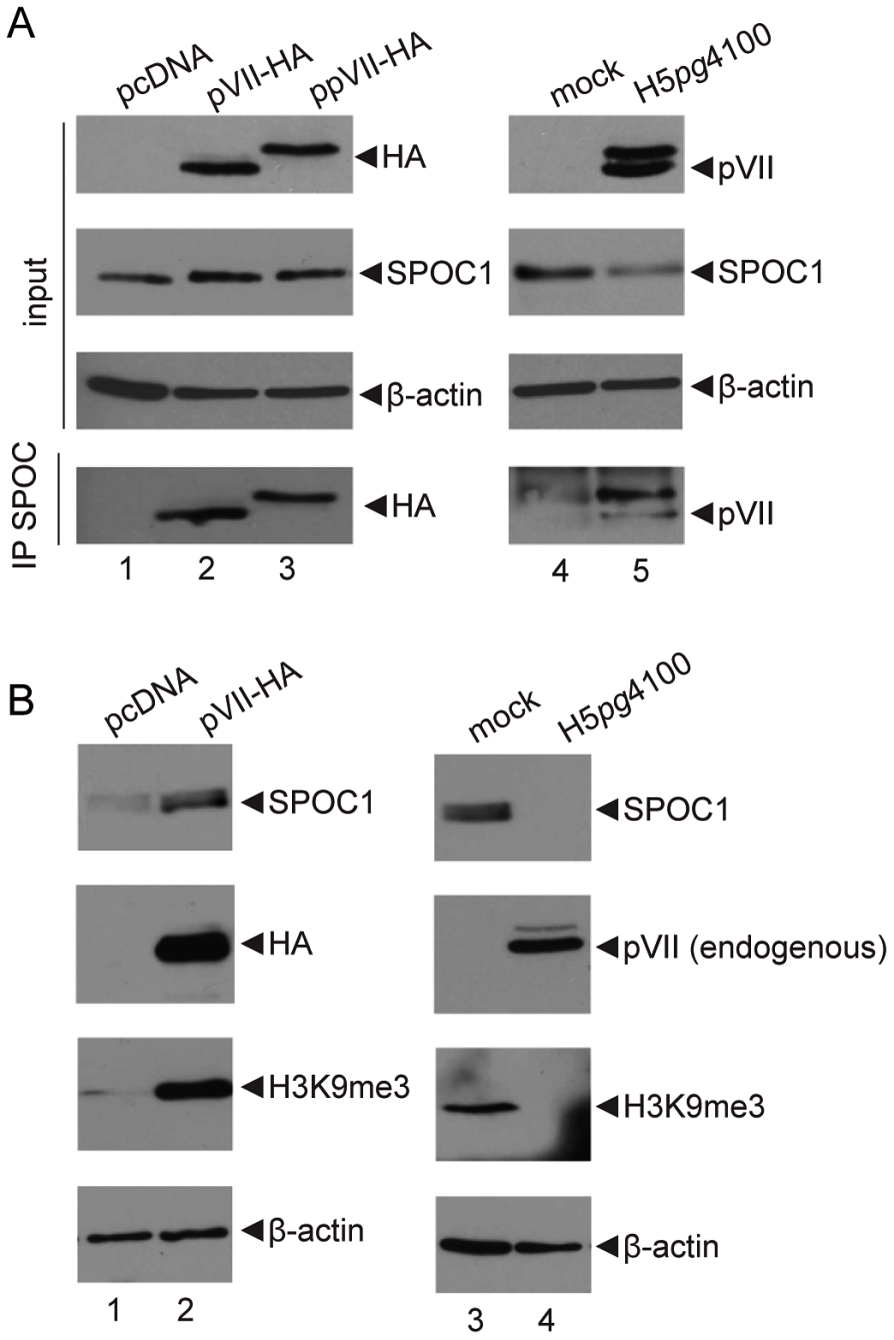
SPOC1 functionally cooperates with Ad core protein pVII. (A) H1299 cells were transfected with pVII expression constructs (ppVII: prepVII; pVII: processed pVII) and infected with wildtype (H5*pg*4100) at moi of 50 FFU per cell. Total cell extracts were prepared 24 hours post transfection and infection. pVII was immunoprecipitated using anti SPOC1 rabbit polyclonal antibody. Proteins were separated on 10% SDS-PAGE and visualized by immunoblotting. Co-precipitated proteins and input levels of total-cell lysates were detected using rat monoclonal antibody 3F10 (HA), rabbit monoclonal pVII antibody, SPOC1-specific rat monoclonal antibody and mouse monoclonal antibody AC-15 (β-actin) as a loading control. (B) H1299 cells were transfected with expression constructs encoding for the processed form of pVII. Total cell extracts were prepared 48 hours post transfection. Proteins were separated on 10% SDS-PAGE, visualized by immunoblotting and detected using rat monoclonal antibody 3F10 (HA), rabbit poyclonal H3K9me3 antibody, SPOC1-specific rat monoclonal antibody and mouse monoclonal antibody AC-15 (β-actin) as a loading control. (C) H1299 cells were infected with wildtype virus (H5*pg*4100) at moi of 50 FFU per cell. Total cell extracts were prepared 48 hours post transfection and infection. Proteins were separated on 10% SDS-PAGE, visualized by immunoblotting and detected by using rabbit pVII antibody, rabbit poyclonal H3K27me3 antibody, SPOC1-specific rat monoclonal antibody and mouse monoclonal antibody AC-15 (β-actin) as a loading control.

SPOC1 has been previously reported to bind histone H3 and to be capable of promoting repressive epigenetic transitions. Since Ad major core protein VII shares homology with the N-terminal regulatory tail of histone H3 [35], we investigated if SPOC1 may also associate pVII. Whole extracts from H1299 cells that had been transfected with plasmid DNA expressing pVII products (Fig. 8A, lanes 1–3) as well as Ad wild type infected lysates (Fig. 8A, lanes 4 and 5) were immunoprecipitated with SPOC1 specific ab and analyzed by Western blotting using anti-HA mab (Fig. 8A). We detected co-immunoprecipitation of SPOC1 with pVII, when both factors were present (Fig. 8A, lanes 2, 3 and 5).

As core protein pVII remains associated with the viral genome during entry [36], we performed further pVII transfection studies mimicking immediate early phase of infection (Fig. 8B). First, we observed a significant increase in SPOC1 protein levels (Fig. 8B, lane 2); and repressive H3K9me3 histone marks as expected due to other reports [19]; Fig. 8B, lane 2. Next, we carried out additional infection studies with H5*pg*4100 Ad wild type virus infected cells (Fig. 8C). Again, we monitored SPOC1 and H3K9me3 protein levels and detected loss of H3K9me3, most presumably due to SPOC1 reduction by proteasomal degradation via E1B-55K/E4orf6 E3 ubiquitin ligases (Fig. 8C, lane 2). As pVII was reported to occupy Ad DNA during the immediate early phase of infection [36], [37], [38], [39], our results indicate that during early Ad infection, pVII likely protects its genome from SPOC1 mediated repressive silencing, prior to the onset of transcription by pVII removal from the genome [40], [41] including loss of pVII-bound repressive factor SPOC1.

### Downregulation of antiviral host factor SPOC1 is not restricted to Ad5 lytic infection

**Figure 9:**
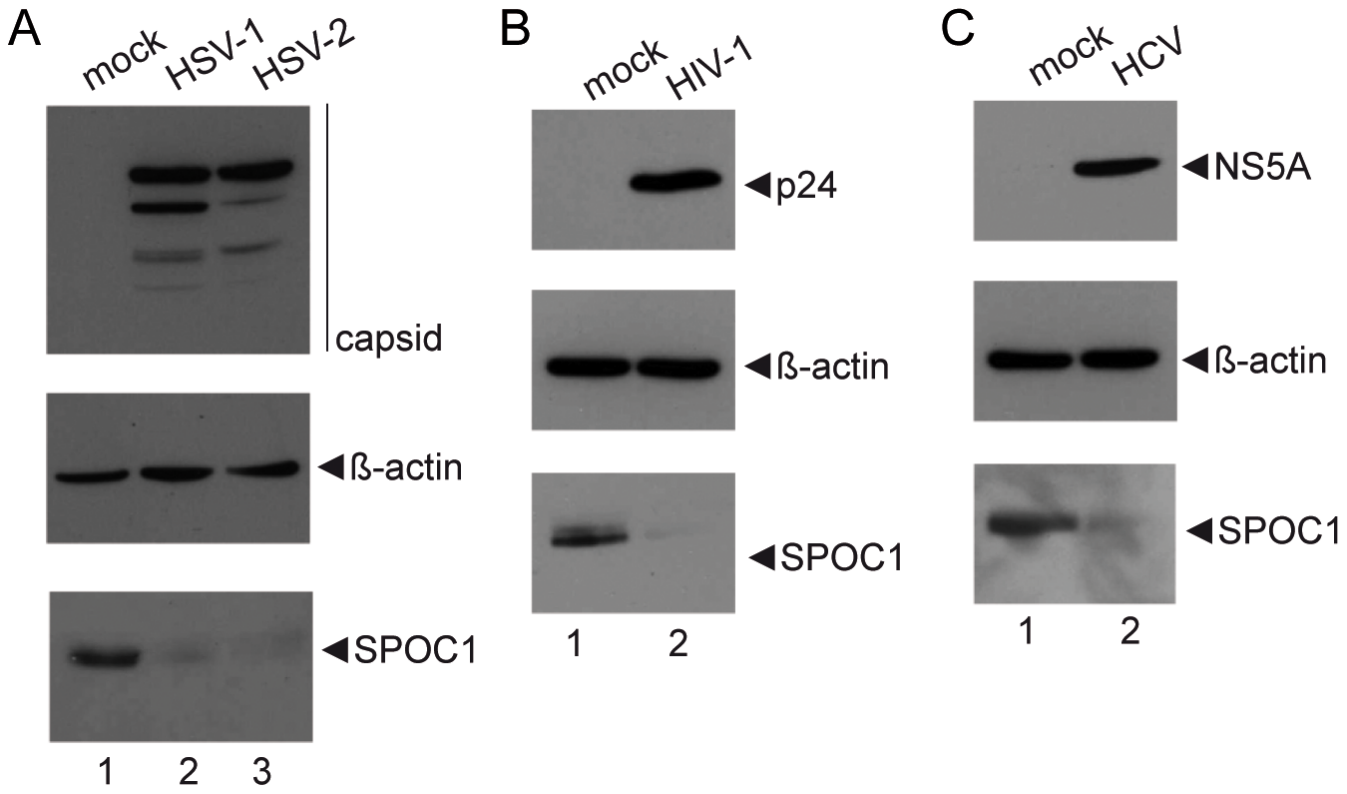
SPOC1 is efficiently reduced during HSV-1, HSV-2, HIV-1 and HCV infection. Permissive cells were infected with (A) HSV-1/HSV-2 (HepaRG cells), (B) HIV-1 (PM-1) and (C) HCV (Huh7.5). HIV-1 infected PM-1 cells (16.3% efficiency) were harvested 4.5 weeks p. i.. HepaRG cells infected with HSV-1 (100% efficiency; moi 2.5 PFU/cell) and HepaRG cells infected HSV-2 (50% efficiency; moi 0.1 PFU/cell) were harvested 18 h p. i.. For HCV infection, Huh7.5 cells were first treated with HCV and then selected with blasticidine. Total cell extracts were prepared and proteins were separated by SDS-PAGE and subjected to immunoblotting using mouse monoclonal antibody 2F6 (NS5A/HCV), p24 hybridoma 183-H12-5C, HSV-1/2 anti-nucleocapsid monoclonal antibody and SPOC1 specific rat monoclonal antibody. β-actin was included as a loading control.

To determine whether SPOC1 is a key player in antiviral defense in general, and whether strategies to restrain SPOC1 are conserved among different human pathogenic viruses, we tested protein expression in permissive cell lines infected with HSV-1 (*herpes simplex virus type 1*; Fig. 9A), HSV-2 (*herpes simplex virus type 2*; Fig. 9A), HIV-1 (*human immunodeficiency virus type 1*; Fig. 9B) or HCV (*human hepatitis C virus*; Fig. 9C). To evaluate efficient infection, we monitored viral protein levels of HSV-1 nucleocapsid protein crossreacting with HSV-2 nuclear protein (Fig. 9A), HIV-1 p24 (Fig. 9B) or HCV NS5A (Fig. 9C). Intriguingly, we detected significantly reduced SPOC1 protein levels in all virus-infected cells (Fig. 9). Taken together, functional inhibition of the antiviral host factor SPOC1 by Ad early proteins E1B-55K and E4orf6 can also be achieved by other viral factors expressed in the course of HSV-1, HSV-2, HIV-1 and HCV infection.

## Discussion

Viruses exploit cellular pathways for their own benefit, often achieved by providing high affinity binding sites on viral factors that recruit key regulatory proteins from cellular pathways to outcompete their physiological binding partner. This strategy allows viruses to infect cells and establish efficient replication with nothing more than the incoming viral genome and viral capsids. Besides transport of the genome to appropriate replication sites, viruses also need to assure decondensation and transcriptional activation of the viral genome, which in most cases has been packed and stored in the most economical way. Once the viral genome becomes transcriptionally activated new viral proteins are synthesized providing the virus with the capacity to reprogram the cell for viral replication.

All of the very early steps during viral infection represent an essential moment in establishing productive infection of all human pathogenic viruses, but are as yet poorly characterized. In this report, we show that the cellular protein SPOC1 is tightly regulated in the course of productive Ad infection, identifying SPOC1 as a antiviral restriction factor in cellular defense. Ad possesses strategies to neutralize SPOC1 via an E1B-55K/E4orf6-dependent proteasomal degradation pathway. Moreover, functional inhibition of SPOC1 seems to be conserved among different human pathogenic viruses.

**Figure 10:**
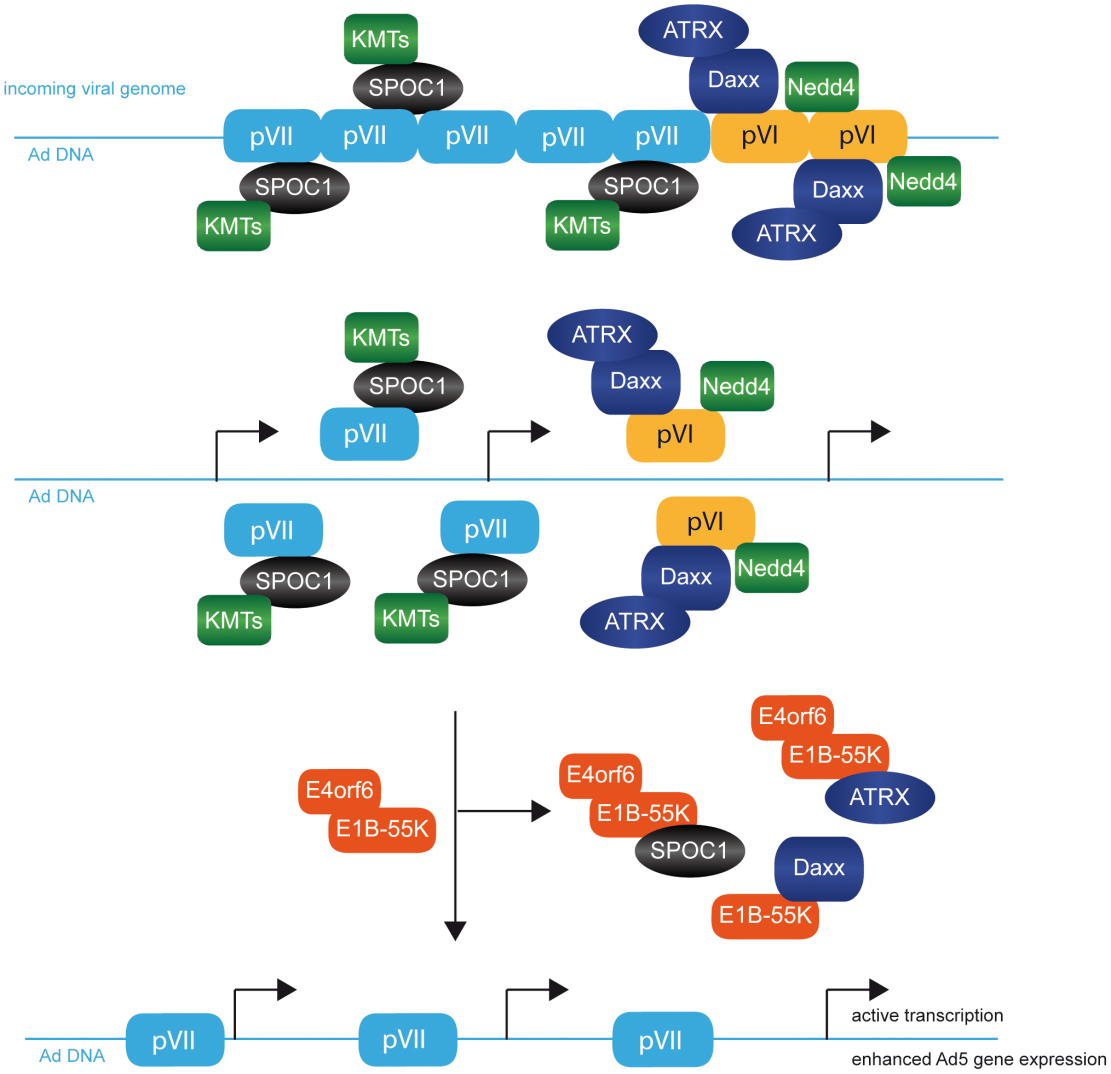
Model for factors involved in early stages after Ad5 virus infection. A schematic representation highlighting the proposed model that pVII recruits SPOC1 to the incoming Ad genome, resulting in pVII-mediated stabilization of SPOC1, followed by its subsequent proteasomal degradation. First, incoming viral DNA is complexed with pV and pVII core/capsid proteins. pVI then mediates interactions with Daxx, ATRX and Nedd4. The pVII/SPOC1 cooperation at viral DNA protects the incoming viral genome from immediate early checkpoint signaling and onset of DNA damage response, resulting in a proviral chromatin microenviroment including KMTs. After activation of viral transcription and E1B-55K/E4orf6 expression, sequestering of Daxx by E1B-55K and E1B-55K/E4orf6 proteolytic degradation of ATRX and SPOC1 host factors promote efficient reduction of repressive histone marks and resulting in active viral transcription and enahnced Ad5 gene expression.

Despite the well-characterized functions of Ad early genes and core/capsid proteins, it is still unclear how Ad transcription is initiated in detail. To summarise our findings we have put together a scheme of how various factors may interact at early stages after Ad infection (Fig. 10). Initially, the genome enters the cell as a highly condensed, transcriptionally inactive nucleoprotein complex, assembled with capsid and core proteins pVI and pVII, identified to recruit cellular factors to the Ad genome Fig. 10; [2], [42]. Recently, we reported transactivating properties of protein VI involving a conserved *PPxY* motif required for binding to ubiquitin ligases of the Nedd4 family of E3 ubiquitin ligases, prior to Ad-dependent depletion of Daxx/ATRX dependent transcriptional restriction Fig. 10; [2].

The Ad major core protein VII remains bound to the Ad genome during the early phase of infection and is subsequently released due to transcription Fig. 10; [37]; however the duration and amount of pVII complexed with the viral genome is still unclear. Moreover, it also remains elusive whether complete disassociation of pVII from viral DNA is required for active transcription. Nevertheless, pVII is the most abundant structural component of the viral core, is strongly associated with viral DNA in a sequence-independent manner [43], and shares homology with the N-terminal regulatory tail of histone H3 [35]. When this viral factor is imported into the nucleus together with the viral genome, it apparently packages the incoming viral DNA into chromatin-like structures Fig. 10; [37], [44], [45], [46], [47].

SPOC1 is a nuclear PHD-protein, predicted to bind H3K4me2/3 and to regulate chromatin-specific interactions [20], [25]. Therefore, SPOC1 is dynamically associated with chromatin, and plays a major role in chromosome condensation to regulate proper cell division [20]. It is proposed that H3K4me2/3-containing chromatin is converted into more compact chromatin by SPOC1-mediated increase of H3K9 KMTs (*lysine methyltransferases*) and H3K9me3 [19].

We observed an association between SPOC1 and viral promoter regions; and with Ad core protein pVII in the absence of any other viral protein, and during Ad infection (Fig. 7B; Fig. 8; Fig. 10). We also observe SPOC1-mediated pVII stabilization, 48 hours post transfection (Fig. 8) and hypothesize that pVII cooperation with viral DNA protects the incoming viral genome from immediate early checkpoint signaling. In accordance with recent observations by Karen and co-workers, we believe that pVII prevents the onset of DNA repair signals prior to efficient viral gene expression [36].

These ideas are further supported by recent evidence that SPOC1 expression levels have a strong impact on DDR, DNA repair, and cellular radiosensitivity [19]. It was observed that SPOC1 and associated factors are recruited to DSBs to modulate chromatin structure as well as DDR. Due to SPOC1 binding and stabilization of repressive factors, i.e. H3K9 KMTs and H3K9me3, this antiviral protein helps conversion into a compact chromatin status (Fig. 10). Conversely, loss of SPOC1 releases several factors and repressive histone marks, promoting chromatin decondensation.

In the early phase of Ad productive infection SPOC1 is recruited to the viral genome and Ad5 replication centers (Fig. 4; Fig. 7B). The SPOC1-rich chromatin environment promotes the activation and recruitment kinetics of DDR transducers (γH2AX, pATM) [19]. However, the Ad core protein pVII masks the genome and antagonizes recognition by host cell repair factors (Fig. 10). Subsequently, core protein VII and pVII bound SPOC1 is released from viral DNA prior to synthesis of Ad replication proteins E2A-DBP, resulting in recognition of viral origins of DNA replication. The replication proteins presumably compete with components of DDR for binding to Ad sequences to form a functional preinitiation complex prior to DDR being inhibited by E1B-55K, E4orf6 and E4orf3.

Interestingly, the correlation between SPOC1 protein levels and H3K9me3 implies that Ad may regulate expression of several H3K9 KMTs. H3K9me3 is not only an epigenetic mark characteristic of heterochromatin, but is also the direct binding platform of cellular and/or viral factors that regulate heterochromatin compaction and spreading [48], [49]. In line with this, Gupta and coworkers recently showed that Ad-dependent E1B-55K/E4orf6 E3 ubiquitin ligase induces proteasomal degradation of the acetyltransferase TIP60 [10]. TIP60 chromodomain binding to H3K9me3 at DSBs is required for its activation [50], and is a prerequisite for efficient ATM activation by acetylation. E1B-55K/E4orf6 might interfere with TIP60 binding to H3K9me3 and its activation, possibly contributing to the observed reduction in activated ATM and delayed DDR.

Furthermore, Ad-mediated SPOC1 (Fig. 1 and 2) and TIP60 depletion would promote chromatin relaxation and decreases accessibility of H3K9me3 for TIP60, leading to reduction in ATM activation. So far, several histone H3K9 KMTs were shown to be modulated by SPOC1 (SETDB1, G9A, GLP) [19]. After the onset of viral transcription and efficient E1B-55K/E4orf6 expression, we observe efficient degradation of SPOC1 host factor (Fig. 1 and 2), promoting efficient reduction of H3K9me3 repressive histone marks (Fig. 8). Our findings provide hints that this complex might be altered in stability and activity due to Ad-dependent proteasomal degradation of SPOC1 (Fig. 1, 2 and 8), since these KMTs form a multi-subunit complex that is destabilized by depleting the individual components [51].

In this report, we show that SPOC1 is tightly regulated in the course of productive Ad infection to neutralize host cell defense processes. SPOC1 represents a novel antiviral restriction factor, being associated with Ad major core protein VII and therefore being removed from the viral genome during the very early phase of infection prior to the onset of gene expression during productive virus life cycle (Fig. 10). In other words SPOC1 apparently plays a biphasic role during Ad life cycle by regulating determinants of chromatin compaction and damage response immediately after viral genome entry into the host nucleus, prior to activation of Ad transcription leading to its proteasomal degradation by newly expressed viral proteins.

Besides Ad, different human pathogenic DNA and RNA viruses can impose reduction on SPOC1 protein levels. Our findings provide a model of how viruses might antagonize intrinsic SPOC1-mediated antiviral responses of their host cells, and create novel awareness for general antiviral restriction factors. These future insights into the immune evasion strategies acquired by viruses and other human pathogens mediated within the host will contribute to identifying new therapeutic strategies and targets to limit or prevent human pathogenic virus-mediated diseases and mortality of patients.

## Materials and Methods

### Cell culture

HEK293 [52], H1299 [53], SPOC1-inducible U2OS and DLD1 cells [19] were grown in Dulbecco's modified Eagle's medium supplemented with 10% fetal calf serum (FCS), 100 U of penicillin and 100 µg of streptomycin per ml in a 5% CO_2_ atmosphere at 37°C. For HepaRG cells, the media was supplemented with 5 µg/ml of bovine insulin and 0.5 µM of hydrocortisone.

### Plasmids, mutagenesis and transient transfections

Ad proteins were expressed from their respective complementary DNAs under the control of the CMV immediate-early promoter, derived from the pcDNA3 vector (*Invitrogen*) to express Ad wild type E1B-55K and E4orf6 [54], [55]. cDNAs encoding E1B-55K-products from each Ad type were cloned as described previously [56], [57], [58]. Transient transfections with luciferase reporter constructs were performed as described previously [2]. pRE-Luc and the RGC-firefly luciferase reporter plasmids pGalTK-Luc have been described previously [59]. SPOC1 wild type protein was expressed from pcDNA4TO-SPOC1 constructs.

### Viruses

H5*pg*4100 served as the wild type Ad5 parental virus in these studies [60]. The mutant viruses H5*pm*4149 and H5*pm*4154 were generated as described recently [61]. Both viruses carry stop codons and do not express the respective viral protein [62]. E4orf6 BC-box mutant virus H5*pm*4139 was generated, resulting in a drastically reduced ability to associate with the Ad5 E3 ubiquitin ligase complex compared to the E4orf6 protein from wild type virus [61]. Viruses were propagated, titrated and infected as described previously [63]. Virus yield was determined by quantitative E2A-72K immunofluorescence staining and viral DNA replication was monitored by quantitative PCR exactly as described previously [13].

### Chromatin Immunoprecipitation Assay (ChIP)

ChIP analysis was performed as described previously [2]. The average C_t_-value was determined from triplicate reactions and normalized with standard curves for each primer pair. The identities of the products obtained were confirmed by melting curve analysis.

### Luciferase reporter assays

For dual luciferase assays, subconfluent cells were transfected prior to preparation of total cell extracts (48 h) [13]. RGC firefly luciferase and pGL-H2A-promoter activity was assayed with lysed extract in an automated luminometer (*Berthold Technologies*). All samples were normalized for transfection efficiency by measuring Renilla-luciferase activity. All experiments shown were performed in triplicate and data are presented as mean values.

### Quantitative real-time (qRT) PCR analysis

Subconfluent cells were infected with wild-type virus and harvested at 24 h p.i.. Total RNA was isolated with *Trizol reagent* (Invitrogen) as described by the manufacturer. The amount of total RNA was measured and one microgram of RNA was reverse transcribed using the *Transcriptor High Fidelity cDNA Synthesis Sample Kit* from Roche including anchored-oligo(dT)_18_ primer specific to the poly(A)^+^RNA. Quantitative real-time PCR was performed with a first strand method in a Rotor-Gene 6000 (Corbett Life Sciences, Sydney, Australia) in 0.5 ml reaction tubes containing a 1/100 dilution of the cDNA template, 10 pmol/µl of each synthetic oligonucleotide primer, 12.5 µl/sample *Power SYBR Green PCR Master Mix* (Applied Biosystems). The PCR conditions were as follows: 10 min at 95°C, 55 cycles of 30 s at 95°C, 30 s at 55 to 62°C (depending upon the primer set) and 30 s at 72°C. The average C_t_ value was determined from triplicate reactions and levels of viral mRNA relative to cellular 18S rRNA were calculated as described recently [13]. The identities of the products obtained were confirmed by melting curve analysis.

### Protein analysis and antibodies (Ab)

For protein analysis cells were resuspended in RIPA buffer as described previously [64]. After 1 h on ice, the lysates were sonicated and the insoluble debris was pelleted at 15,000×g/4°C. For immunoprecipitation and immunoblotting protein lysates were treated as described recently [2]. Primary Ab specific for Ad proteins used in this study included E1B-55K mab 2A6 [65], E2A-72K mouse mab B6-8 [66], E4orf6 mab RSA3 [67], rabbit polyclonal serum against protein VI [68] and anti-pVII rabbit polyclonal antibody (generously provided by Dan Engel, University of Virginia). To evaluate efficient infection with different RNA and DNA viruses primary antibodies specific for HSV-1 nucleocapsid protein (monoclonal mouse mab H1.4; *Acris antibodies*) crossreacting with HSV-2 nuclear protein, HIV-1 p24 hybridoma 183-H12-5C [69] and HCV NS5A (monoclonal mab 2F6/G11 from *immunological and biochemical test systems*) were used.

Primary antibodies specific for cellular proteins included SPOC1 rabbit polyclonal CR56 and rat mab [20], rabbit polyclonal ab specific for histone variant H3K9me3 (*Upstate*), Mre11 rabbit polyclonal antibody pNB 100–142 (*Novus Biologicals, Inc*.), p53 rabbit ab FL393 (*Santa Cruz Biotechnology, Inc.* [70]), polyclonal rabbit antibody raised against SAF-A protein [71] and ß-actin mouse mab AC-15 (*Sigma-Aldrich, Inc.*). HA-epitopes were detected with rat monoclonal 3F10 (*Roche*). Secondary Ab conjugated to horseradish peroxidase (HRP) to detect proteins by immunoblotting were anti-rabbit IgG, anti-rat IgG and anti-mouse IgG (*Jackson/Dianova*).

### Indirect immunofluorescence

Cells were prepared and analyzed as described recently [57]. Images were cropped using Adobe Photoshop CS4 and assembled with Adobe Illustrator CS4.

## Supporting Information

Figure S1 DLD1 and U2OS cells were seeded and treated with doxycyclin to induce SPOC1 expression. The number of living cells was monitored at 1, 3, 5 and 6 days post plating by trypane blue staining to determine the number of cells growing in the absence or presence of doxycyclin.
(TIF)

Figure S2 H1299 cells were infected with wildtype (H5*pg*4100) and mutant viruses (H5*pm*4149, H5*pm*4154) at moi of 50 FFU per cell. Total-cell extracts were prepared 48 hours post infection. E2A/DBP and E1B-55K were immunoprecipitated using rabbit polyclonal SPOC1 antibody. Proteins were separated on 10% SDS-PAGE and visualized by immunoblotting. Input levels of total-cell lysates and co-precipitated proteins were detected using monoclonal antibody B6-8 (E2A/DBP), 2A6 (E1B-55K), SPOC-1-specific rat monoclonal antibody, and mouse monoclonal antibody AC-15 (β-actin) as a loading control.
(TIF)

Figure S3 U2OS cells were seeded and treated with doxycyclin to induce SPOC1 expression. After 12 hours U2Os cells were infected with H5*pg*4100 at a MOI of 800 FFU/cell, fractionated at 20 min intervals and subjected to SDS PAGE using SPOC1 specific rat monoclonal antibody, serum against protein VI, polyclonal antibody against the splicing factor SAF-A (nuclear fraction) as indicated to the right.
(TIF)

Figure S4 H1299 cells were transfected with 0.5 µg of p*Renilla*-Luc, 0.5 µg pH2A-promoter and 0.5 µg pE2F-1, pcDNA4TO-SPOC1 in the combinations indicated (+). Absolute *Firefly*-luciferase activity is shown with mean and standard deviations from three independent experiments.
(TIF)

## References

[1] Blanchette P, Branton PE (2009) Manipulation of the ubiquitin-proteasome pathway by small DNA tumor viruses. Virology 384: 317–323. 19013629 10.1016/j.virol.2008.10.005

[2] Schreiner S, Martinez R, Groitl P, Rayne F, Vaillant R, et al (2012) Transcriptional activation of the adenoviral genome is mediated by capsid protein. PLoS Pathog 8: e1002549. 22427750 10.1371/journal.ppat.1002549 PMC3303589

[3] Berk AJ (2005) Recent lessons in gene expression, cell cycle control, and cell biology from adenovirus. Oncogene 24: 7673–7685. 16299528 10.1038/sj.onc.1209040

[4] Berscheminski J, Groitl P, Dobner T, Wimmer P, Schreiner S (2012) The Adenoviral Oncogene E1A-13S Interacts with a Specific Isoform of the Tumor Suppressor PML To Enhance Viral Transcription. J Virol 87: 965–977. 23135708 10.1128/JVI.02023-12 PMC3554061

[5] Blackford AN, Grand RJ (2009) Adenovirus E1B 55-kilodalton protein: multiple roles in viral infection and cell transformation. J Virol 83: 4000–4012. 19211739 10.1128/JVI.02417-08 PMC2668481

[6] Schreiner S, Wimmer P, Dobner T (2012) Adenovirus degradation of cellular proteins. Future Microbiol 7 2: 211–225. 22324991 10.2217/fmb.11.153

[7] Harada JN, Shevchenko A, Pallas DC, Berk AJ (2002) Analysis of the adenovirus E1B-55K-anchored proteome reveals its link to ubiquitination machinery. J Virol 76: 9194–9206. 12186903 10.1128/JVI.76.18.9194-9206.2002 PMC136464

[8] Blanchette P, Cheng CY, Yan Q, Ketner G, Ornelles DA, et al (2004) Both BC-box motifs of adenovirus protein E4orf6 are required to assemble an E3 ligase complex that degrades p53. Mol Cell Biol 24: 9619–9629. 15485928 10.1128/MCB.24.21.9619-9629.2004 PMC522240

[9] Querido E, Blanchette P, Yan Q, Kamura T, Morrison M, et al (2001) Degradation of p53 by adenovirus E4orf6 and E1B55K proteins occurs via a novel mechanism involving a Cullin-containing complex. Genes Dev 15: 3104–3117. 11731475 10.1101/gad.926401 PMC312842

[10] Gupta A, Jha S, Engel DA, Ornelles DA, Dutta A (2012) Tip60 degradation by adenovirus relieves transcriptional repression of viral transcriptional activator EIA. Oncogene 32: 5017–25. 23178490 10.1038/onc.2012.534 PMC3955737

[11] Blackford AN, Patel RN, Forrester NA, Theil K, Groitl P, et al (2010) Adenovirus 12 E4orf6 inhibits ATR activation by promoting TOPBP1 degradation. Proc Natl Acad Sci U S A 107: 12251–12256. 20566845 10.1073/pnas.0914605107 PMC2901489

[12] Schreiner S, Wimmer P, Sirma H, Everett RD, Blanchette P, et al (2010) Proteasome-dependent degradation of Daxx by the viral E1B-55K protein in human adenovirus-infected cells. J Virol 84: 7029–7038. 20484509 10.1128/JVI.00074-10 PMC2898266

[13] Schreiner S, Bürck C, Glass M, Groitl P, Wimmer P, et al (2013) Control of Human Adenovirus type 5 (Ad5) gene expression by cellular Daxx/ATRX chromatin-associated complexes. Nucleic Acids Res 41: 3532–50. 23396441 10.1093/nar/gkt064 PMC3616723

[14] Araujo FD, Stracker TH, Carson CT, Lee DV, Weitzman MD (2005) Adenovirus type 5 E4orf3 protein targets the Mre11 complex to cytoplasmic aggresomes. J Virol 79: 11382–11391. 16103189 10.1128/JVI.79.17.11382-11391.2005 PMC1193610

[15] Evans JD, Hearing P (2003) Distinct Roles of the Adenovirus E4 ORF3 Protein in Viral DNA Replication and Inhibition of Genome Concatenation. J Virol 77: 5295–5304. 12692231 10.1128/JVI.77.9.5295-5304.2003 PMC153982

[16] Evans JD, Hearing P (2005) Relocalization of the Mre11-Rad50-Nbs1 complex by the adenovirus E4 ORF3 protein is required for viral replication. J Virol 79: 6207–6215. 15858005 10.1128/JVI.79.10.6207-6215.2005 PMC1091726

[17] Stracker TH, Carson CT, Weitzman MD (2002) Adenovirus oncoproteins inactivate the Mre11 Rad50 NBS1 DNA repair complex. Nature 418: 348–352. 12124628 10.1038/nature00863

[18] Stracker TH, Lee DV, Carson CT, Araujo FD, Ornelles DA, et al (2005) Serotype-specific reorganization of the Mre11 complex by adenoviral E4orf3 proteins. J Virol 79: 6664–5573. 15890904 10.1128/JVI.79.11.6664-6673.2005 PMC1112111

[19] Mund A, Schubert T, Staege H, Kinkley S, Reumann K, et al (2012) SPOC1 Modulates DNA Repair by Regulating Key Determinants of Chromatin Compaction and DNA Damage Response. Nucleic Acids Res 40: 11363–11379. 23034801 10.1093/nar/gks868 PMC3526275

[20] Kinkley S, Staege H, Mohrmann G, Rohaly G, Schaub T, et al (2009) SPOC1: a novel PHD-containing protein modulating chromatin structure and mitotic chromosome condensation. J Cell Sci 122: 2946–2956. 19638409 10.1242/jcs.047365

[21] Imyanitov EN, Birrell GW, Filippovich I, Sorokina N, Arnold J, et al (1999) Frequent loss of heterozygosity at 1p36 in ovarian adenocarcinomas but the gene encoding p73 is unlikely to be the target. Oncogene 18: 4640–4642. 10467409 10.1038/sj.onc.1202863

[22] Ragnarsson G, Eiriksdottir G, Johannsdottir JT, Jonasson JG, Egilsson V, et al (1999) Loss of heterozygosity at chromosome 1p in different solid human tumours: association with survival. Br J Cancer 79: 1468–1474. 10188892 10.1038/sj.bjc.6690234 PMC2362732

[23] Mohrmann G, Hengstler JG, Hofmann TG, Endele SU, Lee B, et al (2005) SPOC1, a novel PHD-finger protein: association with residual disease and survival in ovarian cancer. Int J Cancer 116: 547–554. 15825179 10.1002/ijc.20912

[24] Bordlein A, Scherthan H, Nelkenbrecher C, Molter T, Bosl MR, et al (2011) SPOC1 (PHF13) is required for spermatogonial stem cell differentiation and sustained spermatogenesis. J Cell Sci 124: 3137–3148. 21852425 10.1242/jcs.085936

[25] Chi P, Allis CD, Wang GG (2010) Covalent histone modifications–miswritten, misinterpreted and mis-erased in human cancers. Nat Rev Cancer 10: 457–469. 20574448 10.1038/nrc2876 PMC3262678

[26] Baker A, Rohleder KJ, Hanakahi LA, Ketner G (2007) Adenovirus E4 34k and E1b 55k oncoproteins target host DNA ligase IV for proteasomal degradation. J Virol 81: 7034–7040. 17459921 10.1128/JVI.00029-07 PMC1933317

[27] Orazio NI, Naeger CM, Karlseder J, Weitzman MD (2011) The adenovirus E1b55K/E4orf6 complex induces degradation of the Bloom helicase during infection. J Virol 85: 1887–1892. 21123383 10.1128/JVI.02134-10 PMC3028918

[28] Zantema A, Fransen JA, Davis OA, Ramaekers FC, Vooijs GP, et al (1985) Localization of the E1B proteins of adenovirus 5 in transformed cells, as revealed by interaction with monoclonal antibodies. Virology 142: 44–58. 2932843 10.1016/0042-6822(85)90421-0

[29] Zantema A, Schrier PI, Davis OA, van Laar T, Vaessen RT, et al (1985) Adenovirus serotype determines association and localization of the large E1B tumor antigen with cellular tumor antigen p53 in transformed cells. Mol Cell Biol 5: 3084–3091. 2943983 10.1128/mcb.5.11.3084-3091.1985 PMC369122

[30] Goodrum FD, Shenk T, Ornelles DA (1996) Adenovirus early region 4 34-kilodalton protein directs the nuclear localization of the early region 1B 55-kilodalton protein in primate cells. J Virol 70: 6323–6335. 8709260 10.1128/jvi.70.9.6323-6335.1996 PMC190658

[31] König C, Roth J, Dobbelstein M (1999) Adenovirus type 5 E4orf3 protein relieves p53 inhibition by E1B-55-kilodalton protein. J Virol 73: 2253–2262. 9971808 10.1128/jvi.73.3.2253-2262.1999 PMC104470

[32] Krätzer F, Rosorius O, Heger P, Hirschmann N, Dobner T, et al (2000) The adenovirus type 5 E1B-55k oncoprotein is a highly active shuttle protein and shuttling is independent of E4orf6, p53 and Mdm2. Oncogene 19: 850–857. 10702793 10.1038/sj.onc.1203395

[33] Wienzek S, Roth J, Dobbelstein M (2000) E1B 55-kilodalton oncoproteins of adenovirus types 5 and 12 inactivate and relocalize p53, but not p51 or p73, and cooperate with E4orf6 proteins to destabilize p53. J Virol 74: 193–202. 10590106 10.1128/jvi.74.1.193-202.2000 PMC111528

[34] Blanchette P, Wimmer P, Dallaire F, Cheng CY, Branton PE (2013) Aggresome Formation by the Adenoviral Protein E1B55K Is Not Conserved among Adenovirus Species and Is Not Required for Efficient Degradation of Nuclear Substrates. J Virol 87: 4872–4881. 23408624 10.1128/JVI.03272-12 PMC3624316

[35] Lee TW, Blair GE, Matthews DA (2003) Adenovirus core protein VII contains distinct sequences that mediate targeting to the nucleus and nucleolus, and colocalization with human chromosomes. J Gen Virol 84: 3423–3428. 14645923 10.1099/vir.0.19546-0

[36] Karen KA, Hearing P (2011) Adenovirus core protein VII protects the viral genome from a DNA damage response at early times after infection. J Virol 85: 4135–4142. 21345950 10.1128/JVI.02540-10 PMC3126275

[37] Chen J, Morral N, Engel DA (2007) Transcription releases protein VII from adenovirus chromatin. Virology 369: 411–422. 17888479 10.1016/j.virol.2007.08.012

[38] Xue Y, Johnson JS, Ornelles DA, Lieberman J, Engel DA (2005) Adenovirus protein VII functions throughout early phase and interacts with cellular proteins SET and pp32. J Virol 79: 2474–2483. 15681448 10.1128/JVI.79.4.2474-2483.2005 PMC546597

[39] Walkiewicz MP, Morral N, Engel DA (2009) Accurate single-day titration of adenovirus vectors based on equivalence of protein VII nuclear dots and infectious particles. J Virol Methods 159: 251–258. 19406166 10.1016/j.jviromet.2009.04.010 PMC2774845

[40] Matsumoto K, Nagata K, Ui M, Hanaoka F (1993) Template activating factor I, a novel host factor required to stimulate the adenovirus core DNA replication. J Biol Chem 268: 10582–10587. 8486711

[41] Okuwaki M, Nagata K (1998) Template activating factor-I remodels the chromatin structure and stimulates transcription from the chromatin template. J Biol Chem 273: 34511–34518. 9852120 10.1074/jbc.273.51.34511

[42] Komatsu T, Haruki H, Nagata K (2011) Cellular and viral chromatin proteins are positive factors in the regulation of adenovirus gene expression. Nucleic Acids Res 39: 889–901. 20926393 10.1093/nar/gkq783 PMC3035442

[43] Wodrich H, Cassany A, D'Angelo MA, Guan T, Nemerow G, et al (2006) Adenovirus core protein pVII is translocated into the nucleus by multiple import receptor pathways. J Virol 80: 9608–9618. 16973564 10.1128/JVI.00850-06 PMC1617226

[44] Brown DT, Westphal M, Burlingham BT, Winterhoff U, Doerfler W (1975) Structure and composition of the adenovirus type 2 core. J Virol 16: 366–387. 1152144 10.1128/jvi.16.2.366-387.1975 PMC354676

[45] van Oostrum J, Burnett RM (1985) Molecular composition of the adenovirus type 2 virion. J Virol 56: 439–448. 4057357 10.1128/jvi.56.2.439-448.1985 PMC252598

[46] Vayda ME, Flint SJ (1987) Isolation and characterization of adenovirus core nucleoprotein subunits. J Virol 61: 3335–3339. 3625842 10.1128/jvi.61.10.3335-3339.1987 PMC255921

[47] Sergeant A, Tigges MA, Raskas HJ (1979) Nucleosome-like structural subunits of intranuclear parental adenovirus type 2 DNA. J Virol 29: 888–898. 448800 10.1128/jvi.29.3.888-898.1979 PMC353248

[48] Maison C, Almouzni G (2004) HP1 and the dynamics of heterochromatin maintenance. Nat Rev Mol Cell Biol 5: 296–304. 15071554 10.1038/nrm1355

[49] Groner AC, Meylan S, Ciuffi A, Zangger N, Ambrosini G, et al (2010) KRAB-zinc finger proteins and KAP1 can mediate long-range transcriptional repression through heterochromatin spreading. PLoS Genet 6: e1000869. 20221260 10.1371/journal.pgen.1000869 PMC2832679

[50] Sun Y, Jiang X, Xu Y, Ayrapetov MK, Moreau LA, et al (2009) Histone H3 methylation links DNA damage detection to activation of the tumour suppressor Tip60. Nat Cell Biol 11: 1376–1382. 19783983 10.1038/ncb1982 PMC2783526

[51] Fritsch L, Robin P, Mathieu JR, Souidi M, Hinaux H, et al (2010) A subset of the histone H3 lysine 9 methyltransferases Suv39h1, G9a, GLP, and SETDB1 participate in a multimeric complex. Mol Cell 37: 46–56. 20129054 10.1016/j.molcel.2009.12.017

[52] Graham FL, Smiley J, Russel WC, Nairn R (1977) Characteristics of a human cell line transformed by DNA from human adenovirus type 5. J Gen Virol 36: 59–72. 886304 10.1099/0022-1317-36-1-59

[53] Mitsudomi T, Steinberg SM, Nau MM, Carbone D, D'Amico D, et al (1992) p53 gene mutations in non-small-lung cell cancer cell lines and their correlation with the presence of ras mutations and clinical features. Oncogene 7: 171–180. 1311061

[54] Nevels M, Täuber B, Spruss T, Wolf H, Dobner T (2001) “Hit-and-run” transformation by adenovirus oncogenes. J Virol 75: 3089–3094. 11238835 10.1128/JVI.75.7.3089-3094.2001 PMC114102

[55] Rubenwolf S, Schütt H, Nevels M, Wolf H, Dobner T (1997) Structural analysis of the adenovirus type 5 E1B 55-kilodalton-E4orf6 protein complex. J Virol 71: 1115–1123. 8995632 10.1128/jvi.71.2.1115-1123.1997 PMC191163

[56] Cheng CY, Gilson T, Dallaire F, Ketner G, Branton PE, et al The E4orf6/E1B55K E3 ubiquitin ligase complexes of human adenoviruses exhibit heterogeneity in composition and substrate specificity. J Virol 85: 765–775. 21068234 10.1128/JVI.01890-10 PMC3020000

[57] Schreiner S, Wimmer P, Groitl P, Chen SY, Blanchette P, et al (2011) Adenovirus Type 5 Early Region 1B 55K Oncoprotein-Dependent Degradation of Cellular Factor Daxx Is Required for Efficient Transformation of Primary Rodent Cells. J Virol 85: 8752–8765. 21697482 10.1128/JVI.00440-11 PMC3165851

[58] Cheng CY, Gilson T, Wimmer P, Schreiner S, Ketner G, et al (2013) The role of E1B55K in E4orf6/E1B55K E3 ligase complexes formed by different human adenovirus serotypes. J Virol 87: 6232–45. 23536656 10.1128/JVI.00384-13 PMC3648088

[59] Nevels M, Rubenwolf S, Spruss T, Wolf H, Dobner T (1997) The adenovirus E4orf6 protein can promote E1A/E1B-induced focus formation by interfering with p53 tumor suppressor function. Proc Natl Acad Sci USA 94: 1206–1211. 9037031 10.1073/pnas.94.4.1206 PMC19769

[60] Groitl P, Dobner T (2007) Construction of adenovirus type 5 early region 1 and 4 virus mutants. Methods Mol Med 130: 29–39. 17401162 10.1385/1-59745-166-5:29

[61] Blanchette P, Kindsmuller K, Groitl P, Dallaire F, Speiseder T, et al (2008) Control of mRNA export by adenovirus E4orf6 and E1B55K proteins during productive infection requires E4orf6 ubiquitin ligase activity. J Virol 82: 2642–2651. 18184699 10.1128/JVI.02309-07 PMC2258987

[62] Kindsmuller K, Groitl P, Hartl B, Blanchette P, Hauber J, et al (2007) Intranuclear targeting and nuclear export of the adenovirus E1B-55K protein are regulated by SUMO1 conjugation. Proc Natl Acad Sci U S A 104: 6684–6689. 17428914 10.1073/pnas.0702158104 PMC1871846

[63] Kindsmuller K, Schreiner S, Leinenkugel F, Groitl P, Kremmer E, et al (2009) A 49-kilodalton isoform of the adenovirus type 5 early region 1B 55-kilodalton protein is sufficient to support virus replication. J Virol 83: 9045–9056. 19587039 10.1128/JVI.00728-09 PMC2738261

[64] Wimmer P, Schreiner S, Everett RD, Sirma H, Groitl P, et al (2010) SUMO modification of E1B-55K oncoprotein regulates isoform-specific binding to the tumour suppressor protein PML. Oncogene 29: 5511–5522. 20639899 10.1038/onc.2010.284

[65] Sarnow P, Sullivan CA, Levine AJ (1982) A monoclonal antibody detecting the adenovirus type 5-E1b-58Kd tumor antigen: characterization of the E1b-58Kd tumor antigen in adenovirus-infected and -transformed cells. Virology 120: 510–517. 7048730 10.1016/0042-6822(82)90054-x

[66] Reich NC, Sarnow P, Duprey E, Levine AJ (1983) Monoclonal antibodies which recognize native and denatured forms of the adenovirus DNA-binding protein. Virology 128: 480–484. 6310869 10.1016/0042-6822(83)90274-x

[67] Marton MJ, Baim SB, Ornelles DA, Shenk T (1990) The adenovirus E4 17-kilodalton protein complexes with the cellular transcription factor E2F, altering its DNA-binding properties and stimulating E1A-independent accumulation of E2 mRNA. J Virol 64: 2345–2359. 2139141 10.1128/jvi.64.5.2345-2359.1990 PMC249396

[68] Wodrich H, Henaff D, Jammart B, Segura-Morales C, Seelmeir S, et al (2010) A capsid-encoded PPxY-motif facilitates adenovirus entry. PLoS Pathog 6: e1000808. 20333243 10.1371/journal.ppat.1000808 PMC2841620

[69] Chesebro B, Wehrly K, Nishio J, Perryman S (1992) Macrophage-tropic human immunodeficiency virus isolates from different patients exhibit unusual V3 envelope sequence homogeneity in comparison with T-cell-tropic isolates: definition of critical amino acids involved in cell tropism. J Virol 66: 6547–6554. 1404602 10.1128/jvi.66.11.6547-6554.1992 PMC240149

[70] Vojtesek B, Bartek J, Midgley CA, Lane DP (1992) An immunochemical analysis of the human nuclear phosphoprotein p53. New monoclonal antibodies and epitope mapping using recombinant p53. J Immunol Methods 151: 237–244. 1378473 10.1016/0022-1759(92)90122-a

[71] Herrmann F, Lee J, Bedford MT, Fackelmayer FO (2005) Dynamics of human protein arginine methyltransferase 1(PRMT1) in vivo. J Biol Chem 280: 38005–38010. 16159886 10.1074/jbc.M502458200

