# Supplementary figures and images for: SPOC1-Mediated Antiviral Host Cell Response Is Antagonized Early in Human Adenovirus Type 5 Infection
Source: PLoS Pathog. 2013 Nov 21;9(11):e1003775. doi: 10.1371/journal.ppat.1003775 (PMC3836738; doi:10.1371/journal.ppat.1003775)

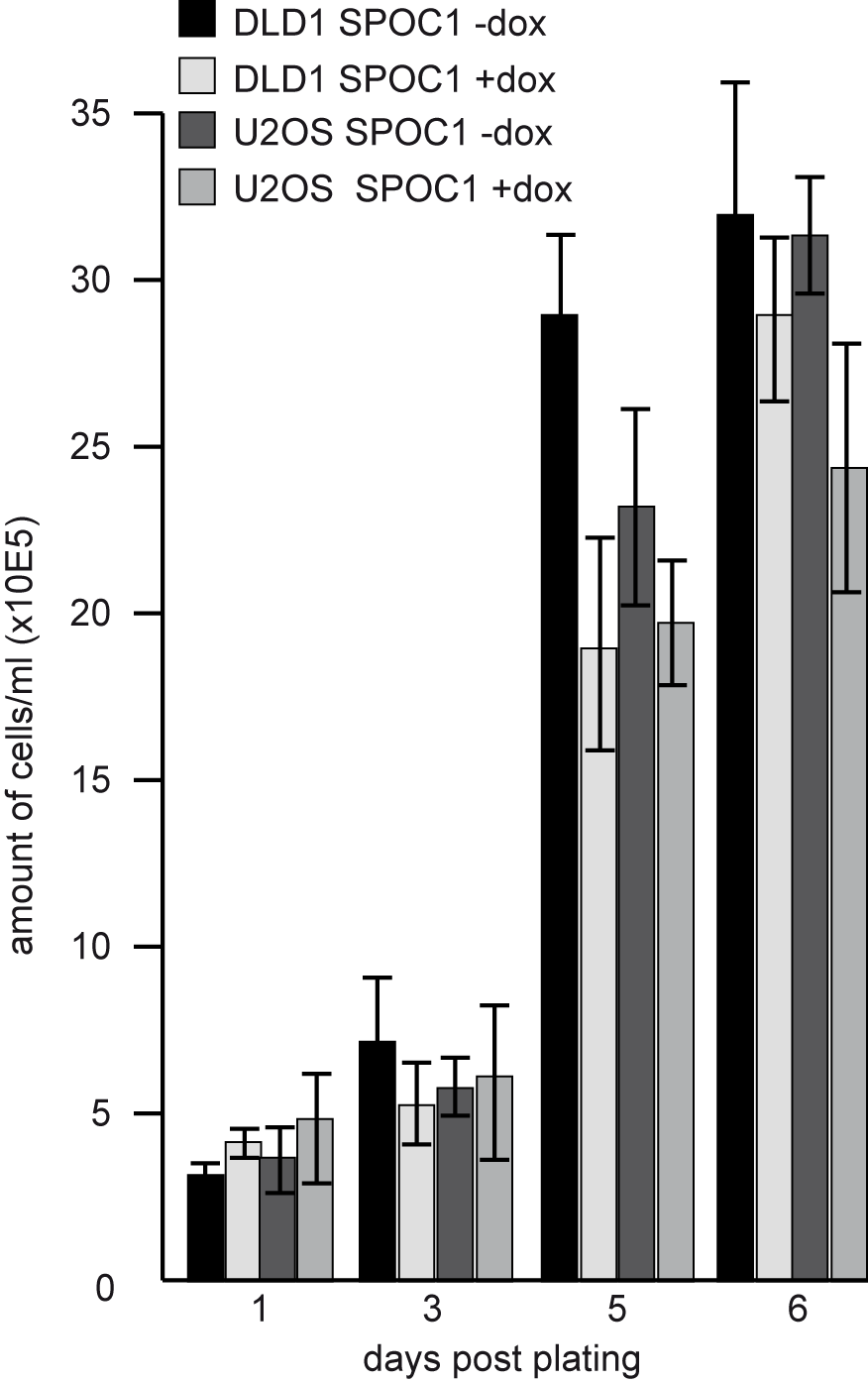

Supplement: Figure S1 — DLD1 and U2OS cells were seeded and treated with doxycyclin to induce SPOC1 expression. The number of living cells was monitored at 1, 3, 5 and 6 days post plating by trypane blue staining to determine the number of cells growing in the absence or presence of doxycyclin. (TIF) [file ppat.1003775.s001.tif]

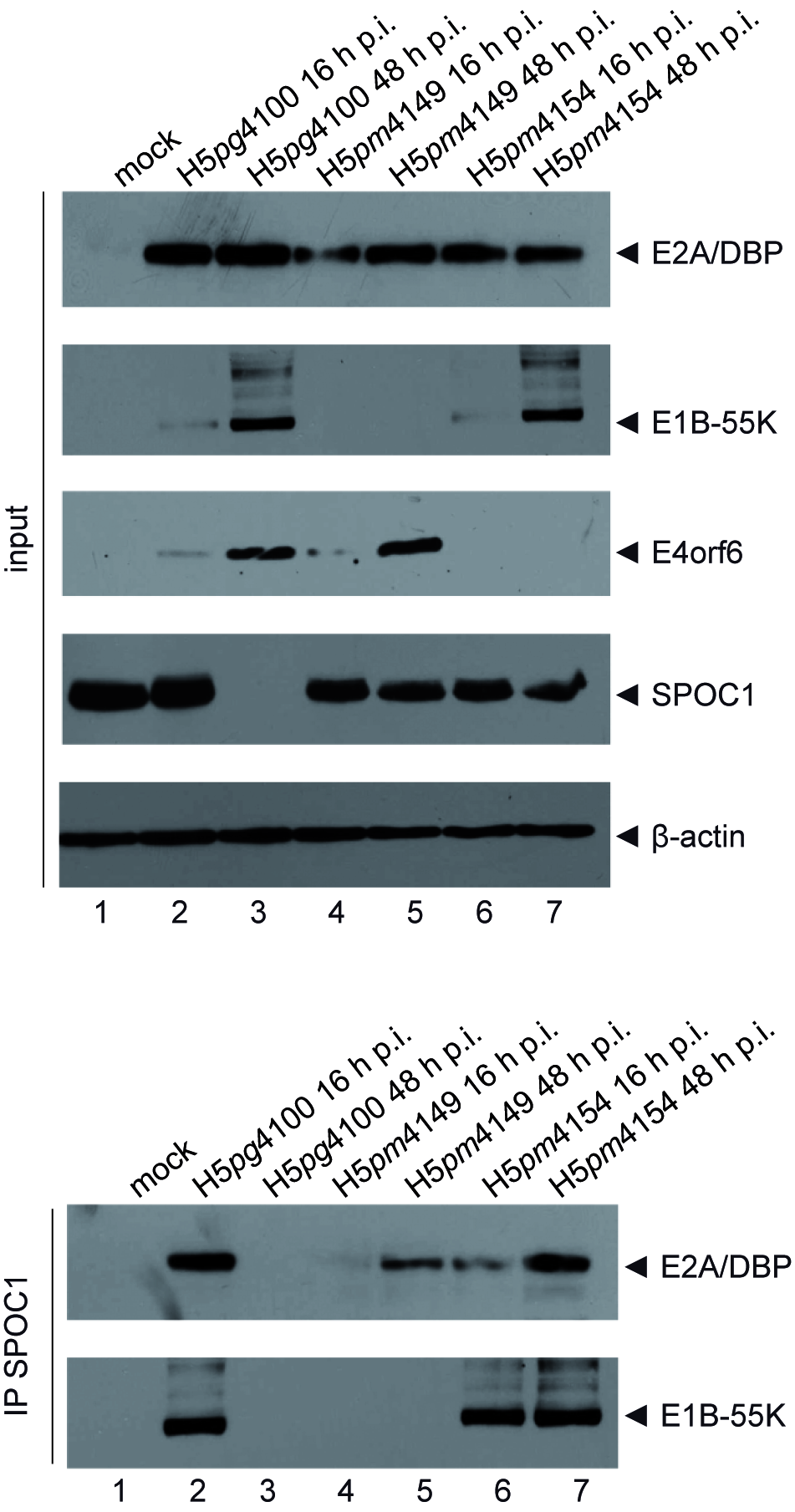

Supplement: Figure S2 — H1299 cells were infected with wildtype (H5pg4100) and mutant viruses (H5pm4149, H5pm4154) at moi of 50 FFU per cell. Total-cell extracts were prepared 48 hours post infection. E2A/DBP and E1B-55K were immunoprecipitated using rabbit polyclonal SPOC1 antibody. Proteins were separated on 10% SDS-PAGE and visualized by immunoblotting. Input levels of total-cell lysates and co-precipitated proteins were detected using monoclonal antibody B6-8 (E2A/DBP), 2A6 (E1B-55K), SPOC-1-specific rat monoclonal antibody, and mouse monoclonal antibody AC-15 (β-actin) as a loading control. (TIF) [file ppat.1003775.s002.tif]

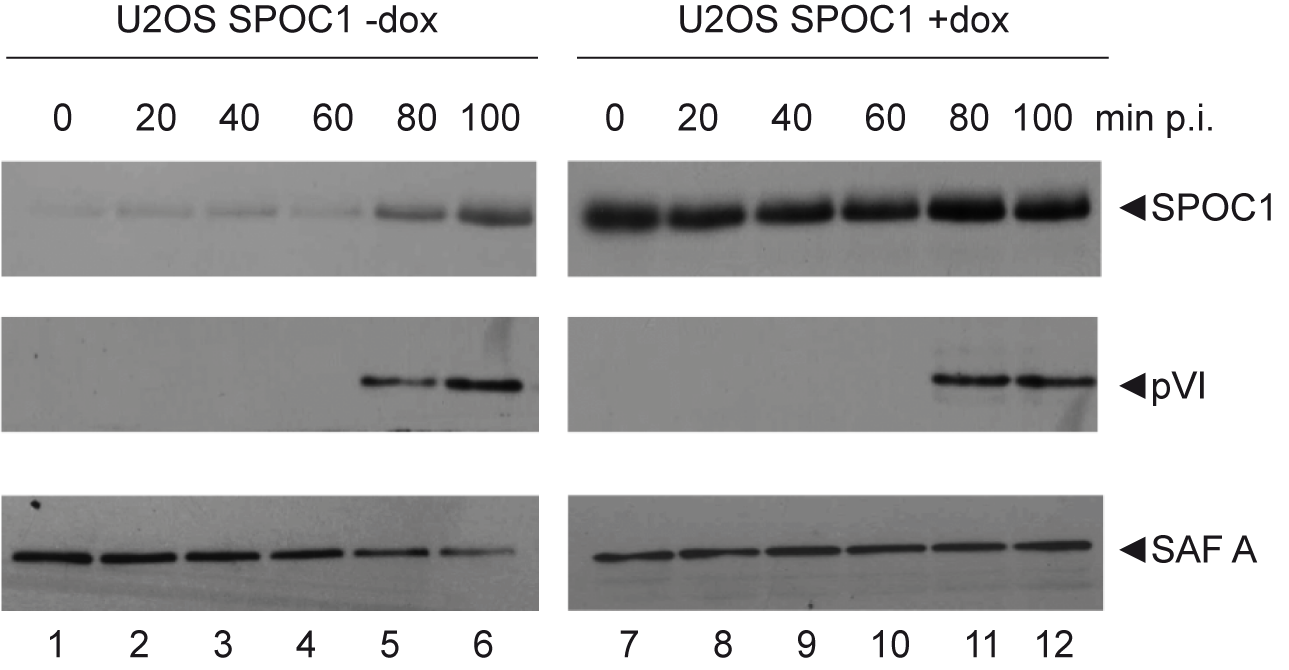

Supplement: Figure S3 — U2OS cells were seeded and treated with doxycyclin to induce SPOC1 expression. After 12 hours U2Os cells were infected with H5pg4100 at a MOI of 800 FFU/cell, fractionated at 20 min intervals and subjected to SDS PAGE using SPOC1 specific rat monoclonal antibody, serum against protein VI, polyclonal antibody against the splicing factor SAF-A (nuclear fraction) as indicated to the right. (TIF) [file ppat.1003775.s003.tif]

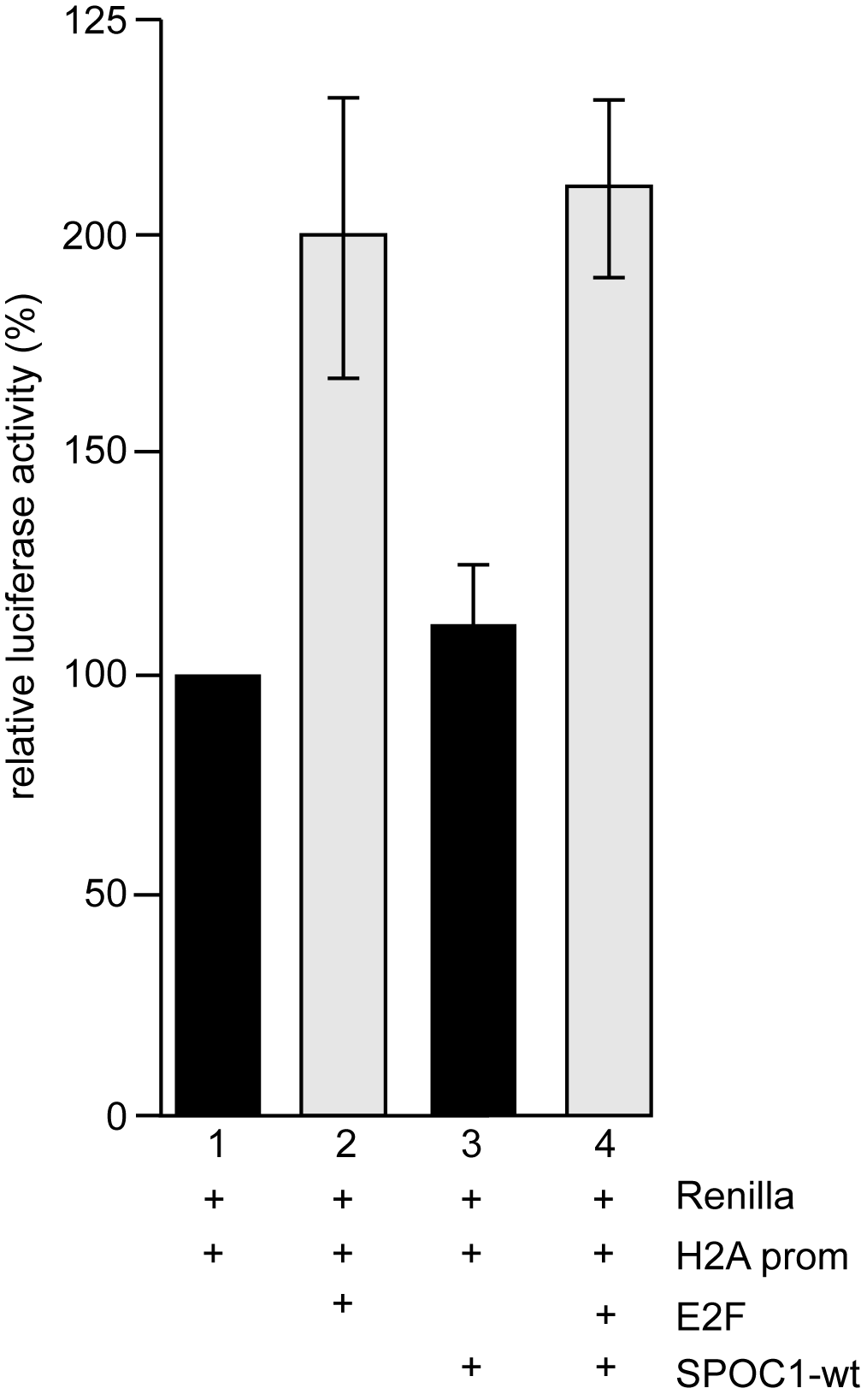

Supplement: Figure S4 — H1299 cells were transfected with 0.5 µg of pRenilla-Luc, 0.5 µg pH2A-promoter and 0.5 µg pE2F-1, pcDNA4TO-SPOC1 in the combinations indicated (+). Absolute Firefly-luciferase activity is shown with mean and standard deviations from three independent experiments. (TIF) [file ppat.1003775.s004.tif]
